# Synchrotron X-ray fluorescence microscopy unveils selenium distribution and a phloem-sink hypothesis in *Neptunia amplexicaulis*

**DOI:** 10.1093/plphys/kiaf367

**Published:** 2025-08-18

**Authors:** Maggie-Anne Harvey, Peter D Erskine, Hugh H Harris, Katherine Pinto-Irish, Daryl L Howard, Melody Fabillo, Antony van der Ent

**Affiliations:** Laboratory of Genetics, Wageningen University and Research, 6708 PB Wageningen, The Netherlands; Centre for Mined Land Rehabilitation, Sustainable Minerals Institute, The University of Queensland, Queensland 4072, Australia; Centre for Mined Land Rehabilitation, Sustainable Minerals Institute, The University of Queensland, Queensland 4072, Australia; Discipline of Chemistry, The University of Adelaide, Adelaide, SA 5005, Australia; Centre for Mined Land Rehabilitation, Sustainable Minerals Institute, The University of Queensland, Queensland 4072, Australia; Australian Synchrotron (ANSTO), XFM Beamline, Clayton, Victoria 3168, Australia; Department of Environment and Science Toowong, Queensland Herbarium and Biodiversity Science, Queensland 4066, Australia; Laboratory of Genetics, Wageningen University and Research, 6708 PB Wageningen, The Netherlands; Centre for Mined Land Rehabilitation, Sustainable Minerals Institute, The University of Queensland, Queensland 4072, Australia

## Abstract

*Neptunia amplexicaulis* is a legume that grows on seleniferous soils in Queensland, Australia. It is one of the strongest known selenium (Se) hyperaccumulators, capable of accumulating up to 13,600 μg Se g^−1^ in its leaves. This study aimed to investigate the distribution and translocation of Se within *N. amplexicaulis* tissues compared to a non-accumulator *Neptunia* species, *Neptunia heliophila*. Synchrotron-based X-ray fluorescence microscopy was used to determine the *in situ* distribution of Se within the organs and tissues of *N. amplexicaulis* and *N. heliophila.* A pulse chase experiment was also undertaken to reveal Se translocation, using a control plant that was analyzed repeatedly after exposure to Se over a 74-h period. Selenium distribution in *N. amplexicaulis* resembled that of *N. heliophila*, albeit at orders of magnitude higher prevailing Se concentrations. In both species, Se concentrations were highest in the youngest/developing plant organs and preferentially localized in the vascular tissues, though Se was also strongly present in the xylem and pith of *N. amplexicaulis*. A phloem-sink model is proposed as the basis of Se distribution in *N. amplexicaulis*. Future studies should focus on elucidating the subcellular distribution of Se and on obtaining insights in the phloem loading and unloading processes of Se.

## Teaser

The selenium hyperaccumulator species *Neptunia amplexicaulis* concentrates high levels of selenium in its phloem sink tissues such as young developing leaves and root tips.

## Introduction

Selenium (Se) hyperaccumulators, plants that uptake and accumulate the element Se in concentrations exceeding 1,000 μg Se g^−1^ within their aerial shoots, were initially discovered growing in regions of the central Western United States with Se-enriched “seleniferous” soils (Beath et al. 1934, 1939). Consumption of these plants by cattle and horses can lead to Se toxicity known as “selenosis”, which has historically been the common method of identifying seleniferous regions and Se hyperaccumulators (Rosenfeld and Beath 1964). Seleniferous soils are defined as any soil > 2 mg Se kg^−1^, though Se has been recorded in concentrations > 100 mg Se kg^−1^ in some locations (Rosenfeld and Beath 1964; Oldfield 2002). Seleniferous soils not only support to hyperaccumulators, but also to plants classified as secondary Se accumulators (with 100 to 1,000 μg Se g^−1^ in their shoots) and non-accumulators (<100 μg Se g^−1^ in their shoots) when growing in their natural habitat (Brown and Shrift 1982; Anderson 1993). Selenium hyperaccumulation occurs in many different species across many families, the genus *Astragalus* (Fabaceae), notably, has 25 different taxa capable of hyperaccumulating Se (Rosenfeld and Beath 1964; White 2016). The two-grooved milkvetch (*Astragalus bisulcatus*) and the cream milkvetch (*Astragalus racemosus*) growing on soils with <10 mg Se kg^−1^, has exceptionally high accumulation exceeding 10,000 μg Se g^−1^ in its shoot tissues (Schiavon and Pilon-Smits 2017). Species of the family Brassicaceae, such as Princes’ Plume (*Stanleya pinnata*) and *Cardamine enshiensis* are also notable Se hyperaccumulators recorded with >4,000 μg Se g^−1^ in their tissues, respectively, where most non-tolerant, non-accumulator plants cannot tolerate in excess of 25 μg Se g^−1^ in their tissues (White et al. 2004; Cappa and Pilon-Smits 2014; White 2016; Both et al. 2020).

### The Australian selenium hyperaccumulator *Neptunia amplexicaulis*

Selenium weed (*Neptunia amplexicaulis* Domin.; Fabaceae), a herbaceous leguminous plant endemic to north-west Queensland (Australia), has been recorded with up to 4,334 μg Se g^−1^ in its shoots in nature (Knott and McCray 1959). Identified as a Se hyperaccumulator as a result of an investigation of cases of cattle selenosis in Richmond, Queensland, the original field studies of *N. amplexicaulis* examined plant Se concentrations in relation to soil Se concentrations over a geographical area; the highest Se plants were found in a particularly highly seleniferous “poison strip” with up to 69 mg Se kg^−1^ in soil (Knott and McCray 1959; McCray and Hurwood 1963). When Se in native soils is lower, concentrations of Se in the plant are also lower, often under the “hyperaccumulation threshold” (McCray and Hurwood 1963). What is not studied in the early field investigations, but examined in later experiments, is the distribution of Se throughout the tissues of this plant species (Harvey et al. 2020; van der Ent et al. 2023). The distribution of Se in organs and tissues was revealed by laboratory X-ray fluorescence-based microscopy (XFM) analysis in plants grown on selenate dosed soil (Harvey et al. 2020), and by using synchrotron-based X-ray fluorescence microscopy (XFM) in specific tissues of plants grown on soil dosed with selenate:selenite solution (van der Ent et al. 2023). This showed that Se occurred in the high concentrations in the youngest and undeveloped leaves (up to 13,600 μg Se g^−1^), while older leaves contained gradually lowering concentrations of Se. Flowers and seed tissues also had relatively high concentrations of Se. Once the leaves matured, Se strongly accumulated in the vascular tissues before translocation to the youngest leaves. Selenium was notably distributed in the phloem of stems and taproot of this species. Due to high prevailing Se concentrations in the root, it was posited that the taproot was the main storage organ for Se in the plant. Additionally, the secondary pulvini (*i.e.* the nodes attaching the compound leaves to the rachis/petiole) were identified as a structure of interest given the presence of Se in this tissue at the base of older leaves. Investigation into the Se distribution of several other hyperaccumulator species have shown similar results, with higher concentrations of Se in the younger leaves compared to mature leaves, and Se concentrations in fruit and floral tissues highest in the plant (Quinn et al. 2011; Lima et al. 2018). Some differences were also noted with Se concentrated in the marginal leaf tissues of *S. pinnata*, and within the leaf lamina surrounding the vascular tissues in *A. racemosus* (Freeman et al. 2006, 2010; van der Ent et al. 2023). Typically, where trichomes are present, they contain no Se (van der Ent et al. 2023).

### The sympatrically occurring comparison species *Neptunia heliophila*

The native sensitive weed (*Neptunia heliophila* A.R. Bean.; Bean 2022; referred to as *Neptunia gracilis* in Harvey et al. 2020 and Pinto Irish et al. 2021) is a plant found growing sympatrically with *N. amplexicaulis*, though it has a far less restricted geographic and edaphic range than the seleniferous species *N. amplexicaulis* (Atlas of Living Australia 2022). It is considered a non-accumulator, recorded with up to 25 μg Se g^−1^ in its shoots from near the “poison strip” and up to 63 μg Se g^−1^ in flowers (McCray and Hurwood 1963; Harvey et al. 2024). It was noted that the distribution of Se within the plant was similar to that of *N. amplexicaulis* but with a less defined gradient between the Se concentrations in younger and slightly older leaves (Harvey et al. 2020). The capacity of *N. heliophila* to uptake and tolerate certain concentrations of Se while also remaining a non-accumulator and its sympatry with a hyperaccumulator from the same genus makes it the ideal comparative system to study the ecophysiology of Se hyperaccumulation.

This study aimed to investigate the distribution of Se within the tissues of hyperaccumulator *N. amplexicaulis* and the related non-accumulator *N. heliophila* using synchrotron-based XFM coupled with Se dosing experiments. Specifically, the *in situ* distribution of Se within tissues, such as the stem, root and leaf, were determined, and finer scale details in fine roots and leaflets were examined. As XFM analysis allows for living plants to be scanned multiple times over without experiencing radiation damage, we aimed to examine Se uptake and translocation using a time-resolved pulse chase experiment in a whole living plant (Blamey et al. 2018). Combining these analytical approaches should advance our understanding of Se distribution and add to the knowledge of Se metabolism in *N. amplexicaulis*.

## Results

### Whole plant biomass across different treatments

**Figure 1.**
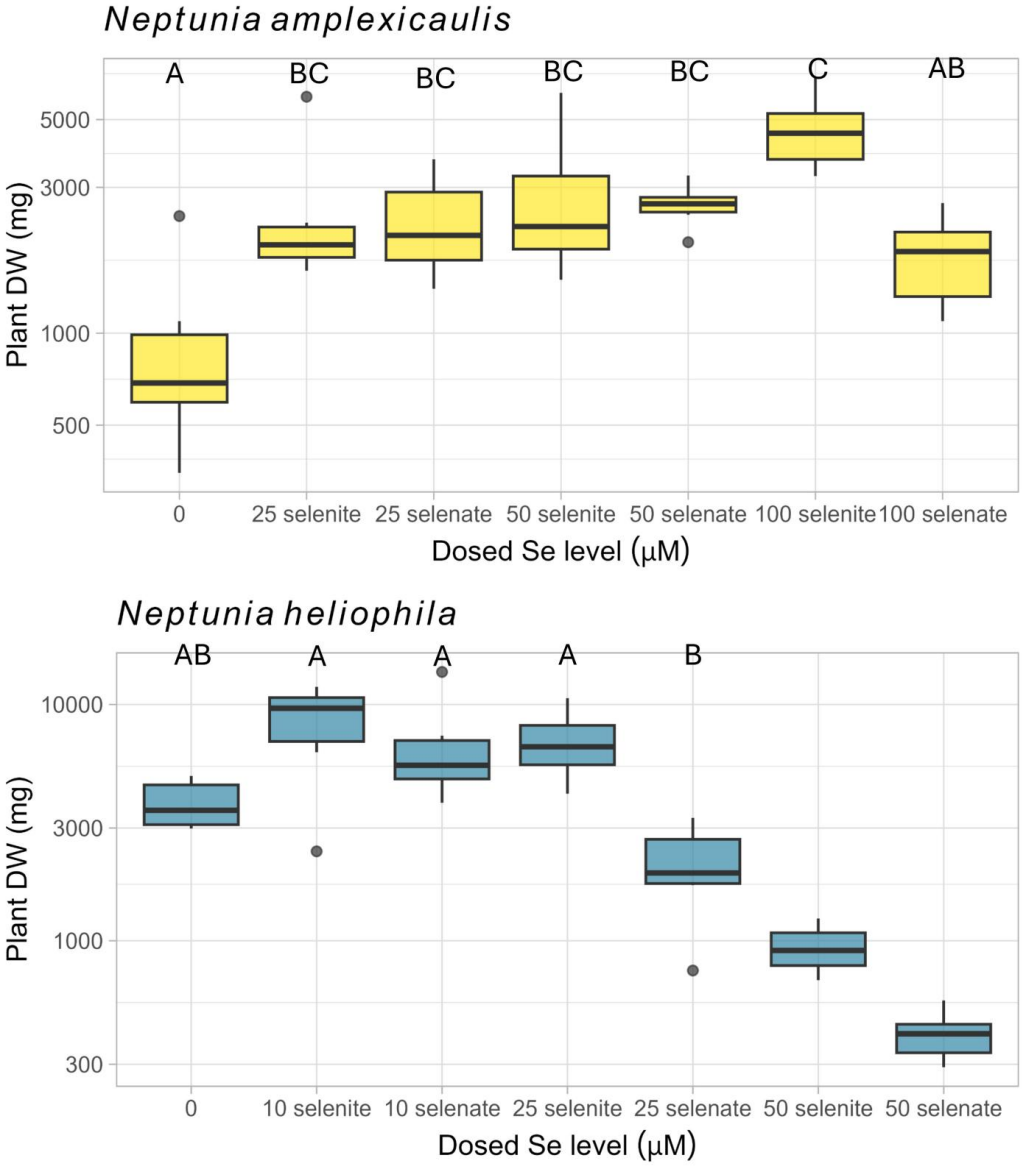
Boxplots showing whole plant biomass dry weight (DW) of *Neptunia amplexicaulis* and *Neptunia heliophila*. Biomass in mg, presented on a log10 scale. Treatments include dosing plants with 0, 10, 25, 50, or 100 µM Se as selenate or selenite. Letters indicate treatments where the plant biomass showed no statistical difference (Tukey's HSD/Dunn's test, *P* < 0.05; Supplementary Tables S1 and S2). Box limits show upper and lower quartiles, centerline shows median, whiskers as ±1.5× interquartile range, circles indicate outliers. For graphing and statistical purposes, half the controls were randomly removed to ensure all treatments had *n* = 6 samples.

Plants grown under a range of Se treatment conditions were examined for differences in whole plant biomass (Fig. 1, Supplementary Tables S1 to S3). In *N. amplexicaulis*, the control plants weighed significantly less (mean is 1,885 mg DW; *P*adj ≤ 0.004; Supplementary Table S3) than all Se treatments, excluding 100 µM selenate treatment (mean is 1,812 mg DW). Comparatively, the 100 µM selenite treated plants weighed the most (mean is 4,685 mg DW), significantly more than the 100 µM selenate plants (*P*adj = 0.007). All other treatments were not statistically different from either of the 100  *µ*M treatments. For *N. heliophila*, however, the plants from the control (mean is 3,963 mg DW) were similar in weight to those from the 10 and 25 µM Se treatments, however the plants from the 25 µM selenate treatment (mean is 2,101 mg DW) were significantly smaller than plants from 10 and 25 µM treatments (*P*adj < 0.001). There appears to be a nonsignificant increase in plant weight in the treatment with the low Se doses, compared to the control (means are 8,476 and 6,806 for 10 µM selenite and selenate plants, respectively; Fig. 1, Supplementary Table S2). The plants from the 50 µM dosed treatments were smaller than those in all other treatments (*P*adj ≤ 0.047), with the 50 µM selenate plants being the smallest (mean is 405 mg DW).

### Whole plant selenium concentrations in bulk tissues

**Figure 2.**
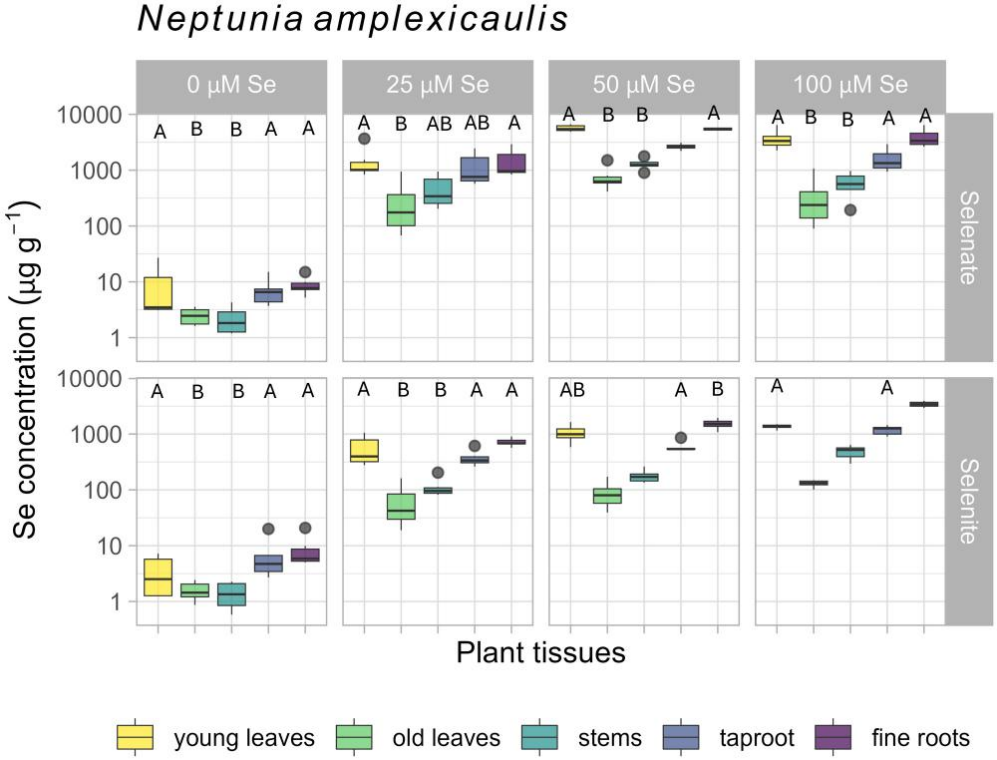
Boxplots showing Se concentrations (log scale, μg g^−1^ dry weight) in bulk tissues of different plant organs in *Neptunia amplexicaulis*, across 8 treatment combinations. Tissues (in color) are young leaves (youngest set of opened and all unopened leaves per stem), old leaves (all other leaves), stems, taproots, and fine roots. Treatments vary in Se level dosed across the top (0, 10, 25, 50, and 100 *μ*M Se) and in the Se form dosed on the left of each graph as selenate (SeO_4_^2−^) or selenite (SeO_3_^2−^). A and B denote groups of statistically similarity of the Se concentration of tissues within treatments, not between treatments (Tukey's HSD/Dunn's test, *P* < 0.05; Supplementary Tables S1 and S2). Box limits show upper and lower quartiles, centerline shows median, whiskers as ±1.5× interquartile range, circles indicate outliers. For 0 μM Se, *n* = 12, all other treatments *n* = 6.

**Figure 3.**
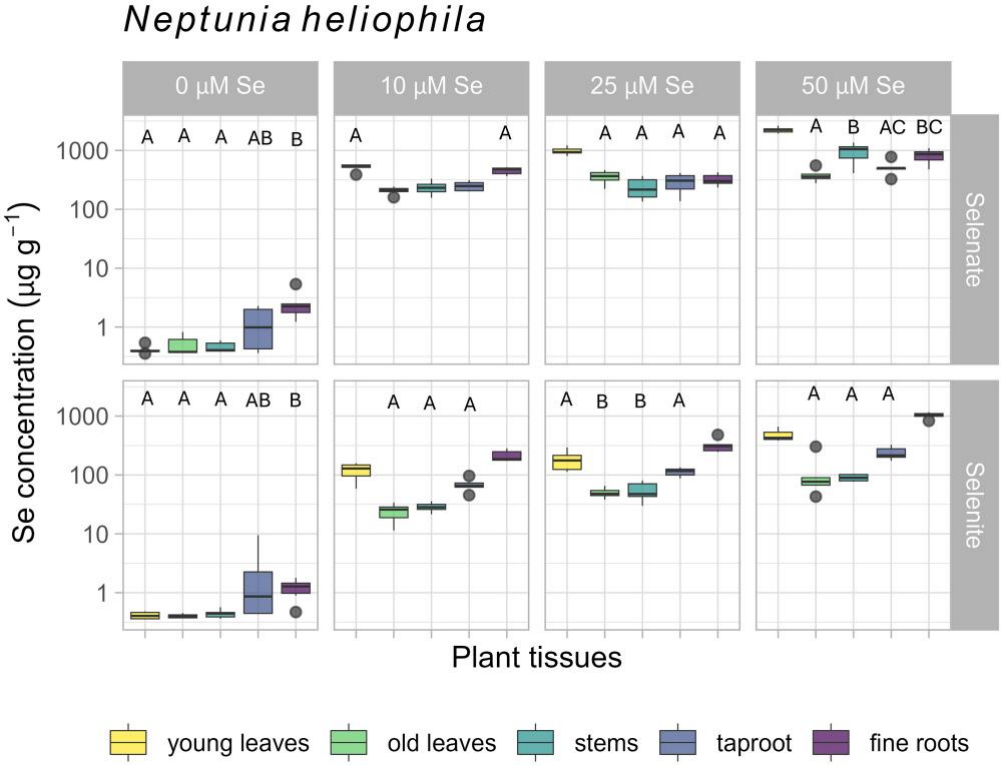
Boxplots showing Se concentrations (log scale, μg g^−1^ DW) in bulk tissues of different plant organs in *Neptunia heliophila*, across 8 treatment combinations. Tissues (in color) are young leaves (youngest set of opened and all unopened leaves per stem), old leaves (all other leaves), stems, taproots, and fine roots. Treatments vary in Se level dosed across the top (0, 10, 25, 50, and 100 μM Se) and in the Se form dosed on the left of each graph as selenate (SeO_4_^2−^) or selenite (SeO_3_^2−^). A and B denote groups of statistically similarity of the Se concentration of tissues within treatments, not between treatments (Tukey's HSD/Dunn's test, *P* < 0.05; Supplementary Tables S1 and S2). Box limits show upper and lower quartiles, centerline shows median, whiskers as ±1.5× interquartile range, circles indicate outliers. For 0 μM Se, *n* = 12, all other treatments *n* = 6.

Plants grown under a range of Se treatment conditions were examined to determine Se distribution ranges between the different plant tissues (Fig. 2; Supplementary Tables S1, S2, and S4). In *N. amplexicaulis*, most commonly the young leaves, taproot, and fine roots were the highest in Se (maximum of 6,631, 2,928, and 6,421 µg Se g^−1^, respectively; Supplementary Table S4), with no statistical difference between these tissues in half the treatments. In three other treatments, there was no difference between the young leaves and fine roots and/or young leaves and taproot (Fig. 2, Supplementary Table S2). The tissues with the lowest concentration of Se were the old leaves then the stems, which showed no statistical difference in Se concentration across the 6 treatments. The control specimens exhibited a similar pattern, accumulating up to 26 µg Se g^−1^ in the young leaves, likely from Se in the seeds collected from the seleniferous field site (Supplementary Table S4). For *N. heliophila* selenate dosed samples, the young leaves were more concentrated in Se than all other tissues (maximum of 2,625 µg Se g^−1^; Fig. 3; Supplementary Table S4), but in one case statistically similar to the fine roots (means 523 to 454 µg Se g^−1^, respectively, in the selenate 10 µM Se treatment). In the selenite treatments, the fine roots were more concentrated in Se than all other tissues (maximum of 1,164 µg Se g^−1^), followed by the young leaves (maximum of 661 µg Se g^−1^). For the control *N. heliophila* specimens, the range of Se concentrations was very low, though there was some detectable Se in the roots (up to 5.35 µg Se g^−1^).

### Elemental distribution in the leaves of *N. amplexicaulis*

**Figure 4.**
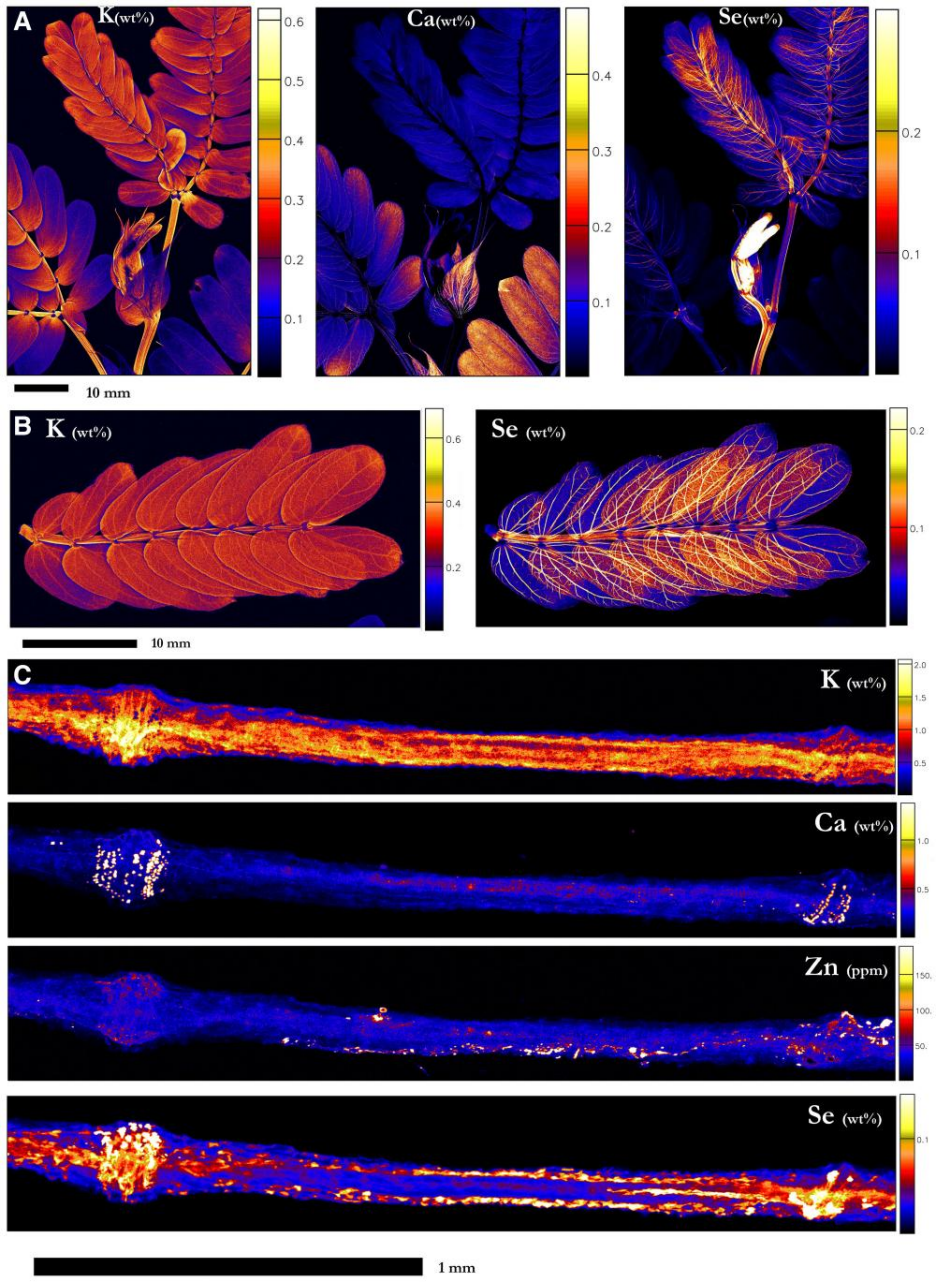
Synchrotron XFM elemental maps showing the distribution of K, Ca, and Se in hydrated leaf sections of *N. amplexicaulis*. **A)** Hydrated leaf and stem portion of *N. amplexicaulis*, **B)** K and Se in a pinna of leaflets, **C)** K, Ca, Zn, and Se in a hydrated leaf cross-section of *N. amplexicaulis.* Plant grown in hydroponics supplied with **A** and **C)** 50:25 *µ*M selenate:selenite treatment (r1, r2) **B)** 75 µM selenate (r1). Scale bars denote M**A** and **B)** 10 mm and **C)** 1,000 µm. Color bars show elemental concentrations in µg g^−1^ (ppm) or weight percent (wt%).

*N. amplexicaulis* exhibited its strongest accumulation of Se in the youngest undeveloped leaves (4,540 µg Se g^−1^), followed by the youngest part of the stem (Fig. 4A; Supplementary Table S5; Supplementary Fig. S1). Lower concentrations of Se were present in the vascular tissues of the youngest more developed leaves, where Se was gradually lower from the vascular tissues of increasingly older leaves (123 µg Se g^−1^ in the oldest leaflet). Selenium was mostly present throughout the vascular tissue of a pinna, more strongly concentrated on the outside of the main arterial vein (Fig. 4B). There was also a lower concentration of Se in the marginal tissue, though it was not detected in the lamina or any of the trichomes. The rachilla contained Se in decreasing concentrations toward the apex, and the pulvini attached the pinna to the rachilla had relatively lower Se concentration. Cross-sections of the younger leaves reveal that Se was strongly concentrated in the vascular bundles (1,150 µg Se g^−1^), and in the epidermis (856 µg Se g^−1^; Fig. 4C; Supplementary Table S6; Supplementary Figs. S1 and S2). Zinc was mainly distributed in the leaf margins, whereas Ca and K were strongly concentrated in the veins. Potassium was also found in considerable concentrations throughout the mesophyll. At the shoot-scale, K was distributed uniformly in the lamina of younger pinna and more concentrated at the basal end of the older leaflets, whereas Ca was more concentrated in the lamina of older pinna than younger (Fig. 4A). The stipules were also more concentrated in Ca, similar to the older leaflets.

### Elemental distribution in leaves of *N. heliophila*

**Figure 5.**
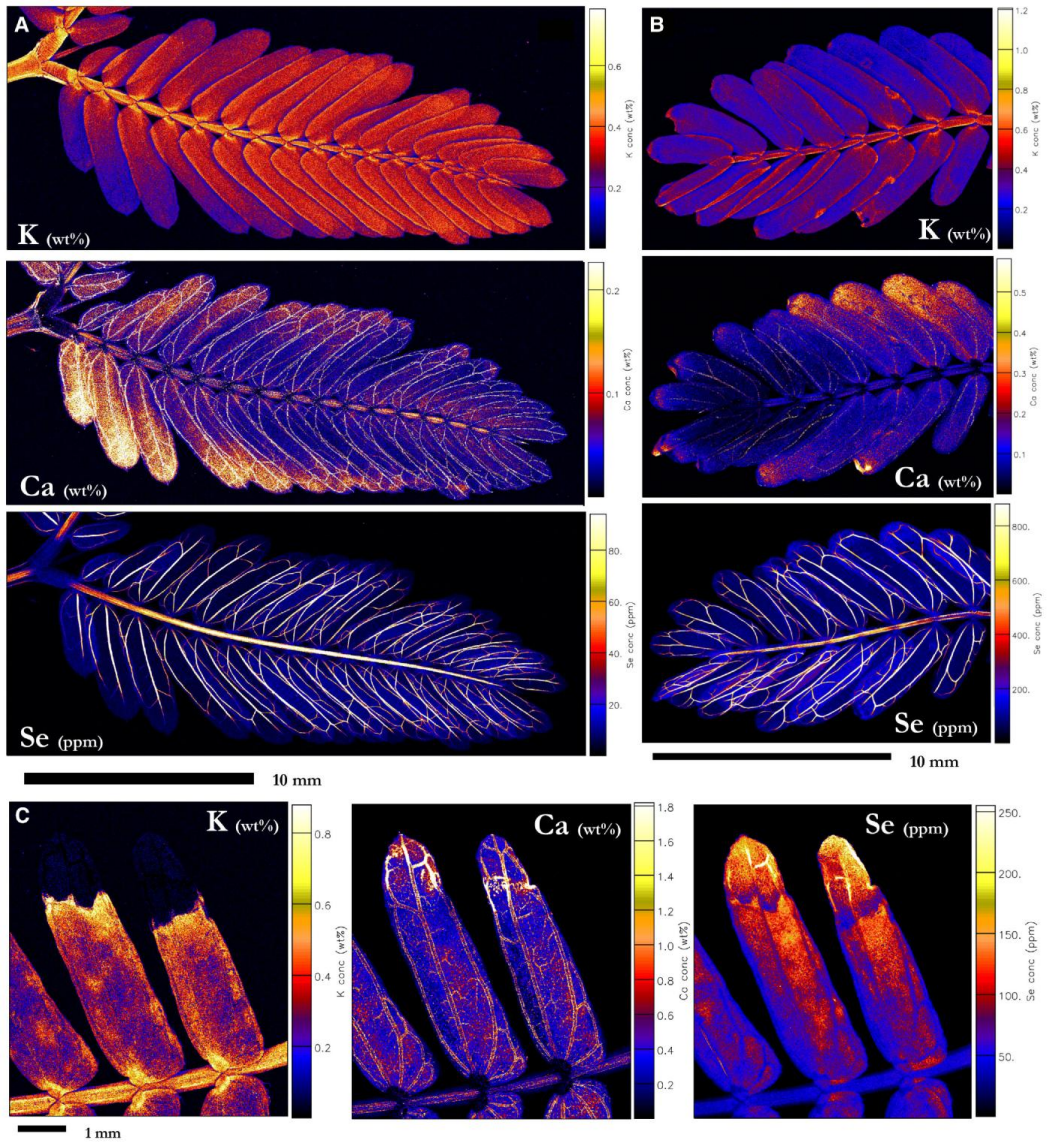
Synchrotron-based XFM elemental maps showing the distribution of K, Ca, and Se in hydrated leaf sections of *N. heliophila.*  **A)** Pinna and secondary pulvini from 25 µM selenite treatment, **B)** pinna from 25 µM selenate treatment (r1), **C)** tip of pinna with leaf damage from Se toxicity from 25 µM selenate treatment (r2). Scale bars denote **A** and **B)** 10 mm and **C)** 1 mm. Color bars show elemental concentrations in µg g^−1^ (ppm) or weight percent (wt%).

The distribution of Se in the leaves of *N. heliophila* was very similar to *N. amplexicaulis*, where Se was confined primarily to the vascular tissues, most concentrated in the undeveloped leaves and youngest section of stem, decreasing in concentration as leaves get older. However, in *N. heliophila*, there was a high concentration of Se in the rachis and rachilla, with concentration increasing toward the tip of the rachilla (Fig. 5). The selenite dosed plants had healthier growth, with Se concentrations in the rachilla and leaf vascular tissues (50 to 100 µg Se g^−1^) exceeding those in the lamina (4.02 µg Se g^−1^) or secondary pulvinus (4 to 8 µg Se g^−1^; Fig. 5A; Supplementary Fig. S3A; Supplementary Table S5). While the same pattern of accumulation was present in the selenate dosed plants, they demonstrated leaf damage and stunting (Fig. 3; Fig. 5C; Supplementary Figs. S1F and S3B). Non-affected leaflets contained 454 µg Se g^−1^ in their vascular tissues (Fig. 5B; Supplementary Table S5), and leaflets with notable deformations and necrosis had variable Se concentrations, up to 620 µg Se g^−1^ in the veins. Selenium was present in the lamina of the more damaged leaflets (115 µg Se g^−1^; Supplementary Fig. S3B), and in another specimen, Se was more concentrated in the necrotic ends (up to 129 µg Se g^−1^; Fig. 5C; Supplementary Fig. S1F).

### Elemental distribution in stems

**Figure 6.**
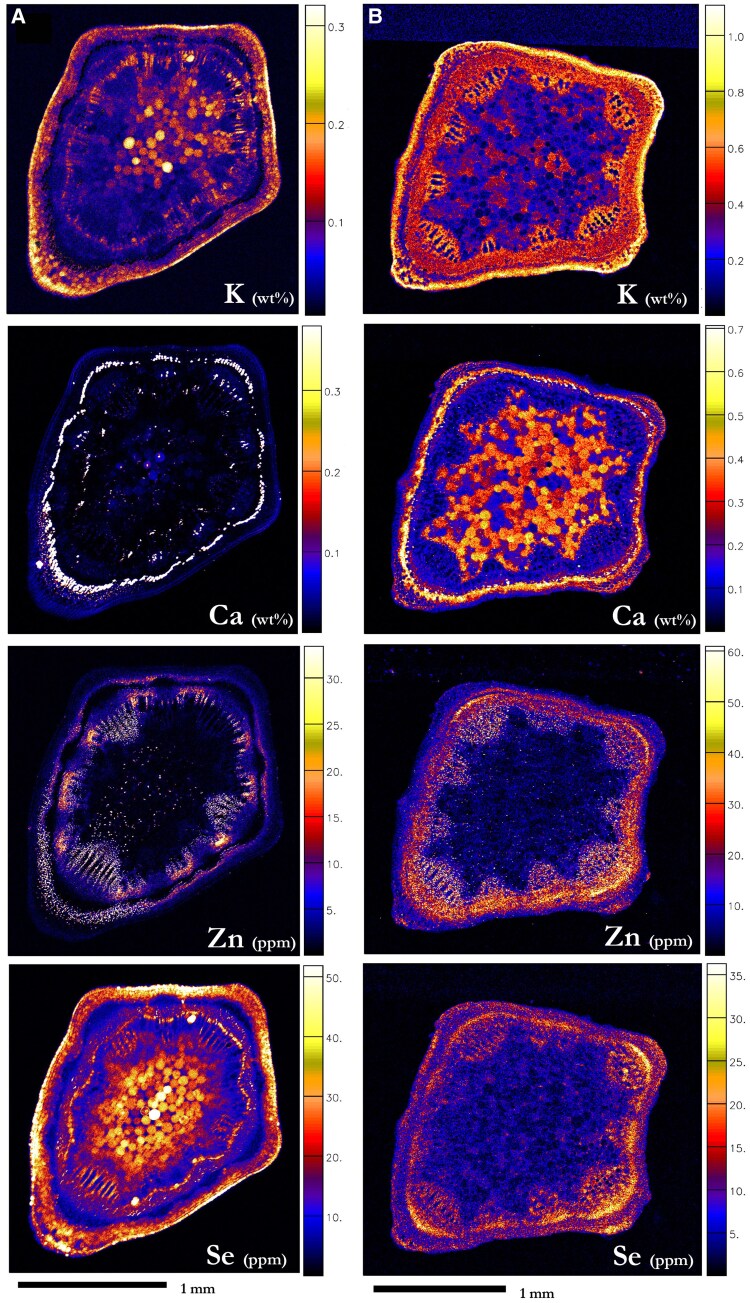
Synchrotron-based XFM elemental maps showing the distribution of K, Ca, Zn, and Se in hydrated stem cross-sections of *Neptunia amplexicaulis* and *N. heliophila.*  **A)**  *N. amplexicaulis* from 50:25 µM selenate:selenite treatment (r3), **B)**  *N. heliophila* from 25 µM selenite treatment (r2). Scale bars denote 1 mm. Color bars show elemental concentrations in µg g^−1^ (ppm) or weight percent (wt%).

The stems of *N. amplexicaulis* contained Se (Fig. 6A; Supplementary Table S6). It was mostly distributed in the cortex (27.3 µg Se g^−1^) and pith (25.1 µg Se g^−1^), followed by the xylem (14.4 µg Se g^−1^) and phloem (up to 16.7 µg Se g^−1^) (Fig. 6A; Supplementary Figs. S1 and S2; Supplementary Table S6). Selenium was relatively lower from the phloem bundles, as were most elements except Ca; however, Se was more concentrated in the phloem closer to the xylem, which was also enriched with higher K and Ca and Zn at times. Zinc was found in the xylem tissues and parts of the phloem in high concentrations. Potassium was highest in the cortex and phloem boundary, and in parts of the epidermis, pith and in small amounts in the phloem and xylem. Calcium was mainly found in the epidermis and structural edges of the cortex, and sections of the pith, though it was in considerably lower concentrations to that observed in the phloem bundles.

The stems of *N. heliophila* contain less Se overall, even at the most concentrated tissues (Fig. 6B; Supplementary Figs. S1 and S2; Supplementary Table S6). Selenium was in higher concentrations in the phloem (13.6 *µ*g Se g^−1^), parts of the epidermis, cortex (10.5 *µ*g Se g^−1^), and xylem (9.64 µg Se g^−1^), but the Se distribution in these tissues was more diffuse when compared to *N. amplexicaulis*. Only minimal Se was present in the pith (4.77 *µ*g Se g^−1^). Zinc distribution closely mimics Se in *N. heliophila* stems when compared with *N. amplexicaulis* stems—it was primarily concentrated in the xylem and phloem, with small amounts also occurring across the cortex and residual procambium. Calcium and K were present throughout the tissues. Calcium was highly concentrated in the pith and phloem, and present structurally in the cortex and epidermis edges, while K was concentrated in the cortex and vascular tissues.

### Elemental distribution in roots

**Figure 7.**
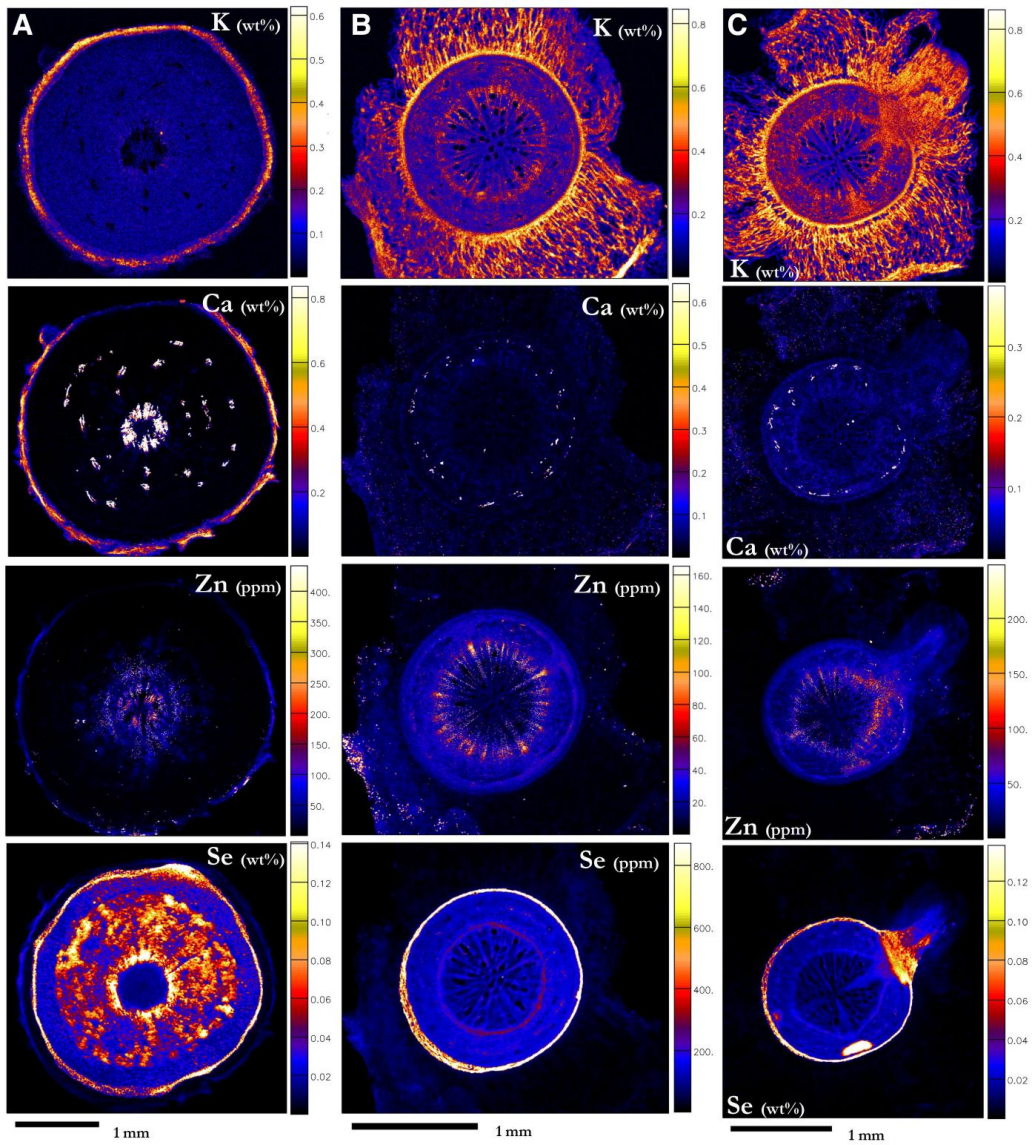
Synchrotron-based XFM elemental maps showing the distribution of K, Ca, Zn, and Se in hydrated root cross-sections of *Neptunia amplexicaulis*. **A)** Older root grown in Se-enriched soil, **B)** root grown in hydroponics, **C)** root grown in hydroponics with emerging lateral root. *Neptunia amplexicaulis* grown in hydroponics supplied with 75 µ*M* selenate treatment (r1). Scale bars denote 1 mm. Color bars show elemental concentrations in µg g^−1^ (ppm) or weight percent (wt%).

**Figure 8.**
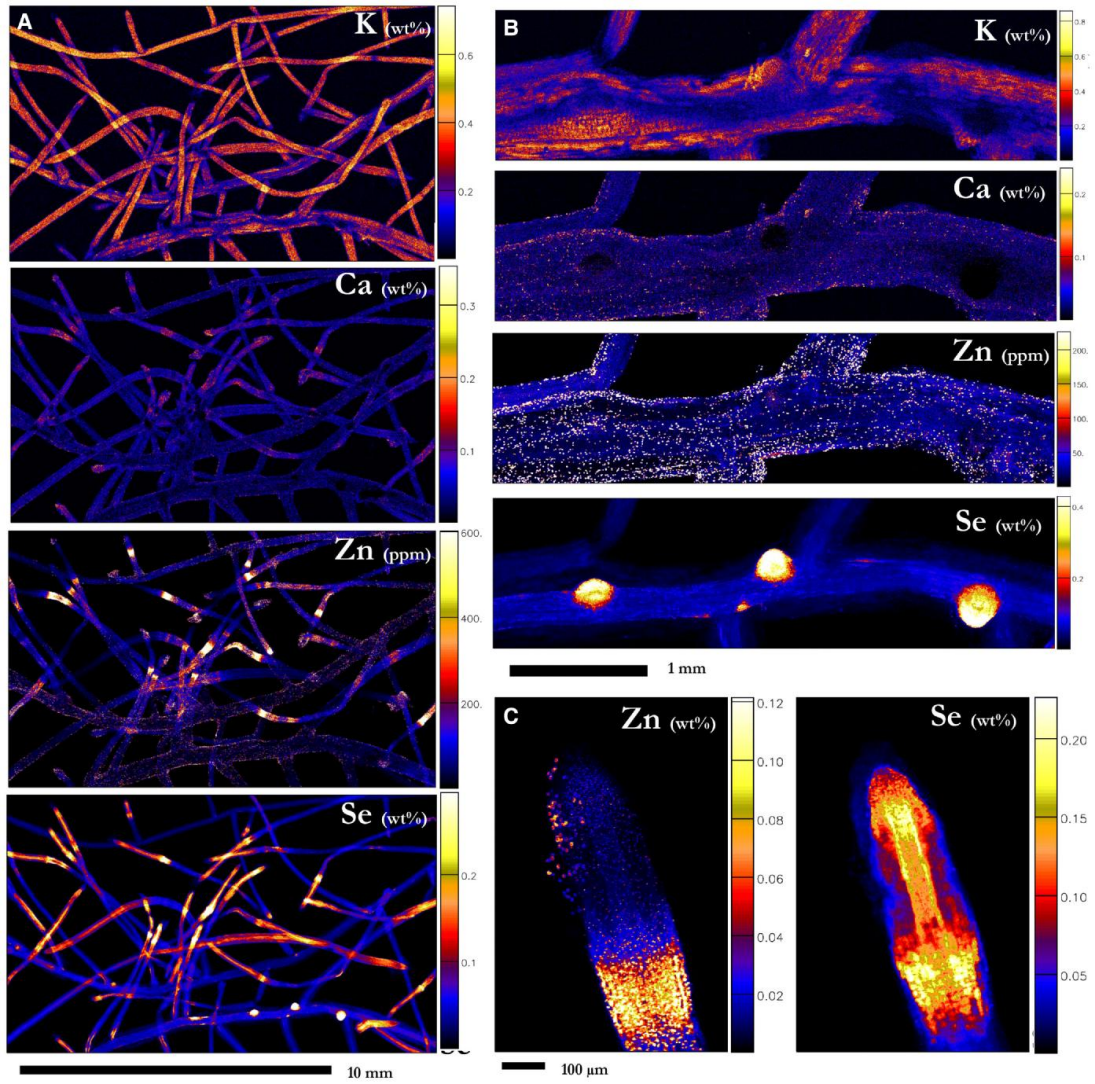
Synchrotron-based XFM elemental maps showing the distribution of K, Ca, Zn, and Se in hydrated fine lateral roots of *N. amplexicaulis*. **A)** A group of fine lateral roots **B)** shows a close-up of newly developing fine roots and **C)** shows a close-up of the fine root tip. Hydroponically grown plants supplied with 75 µM selenate treatment (r2). Scale bars denote **A)** 10 mm, **B)** 1 m, and **C)** 100 µm. Color bars show elemental concentrations in µg g^−1^ (ppm) or weight percent (wt%).

Older roots from plants grown in soil, exhibiting secondary growth characteristics, contained Se heterogeneously in the secondary xylem (583 µg Se g^−1^), and at very high concentrations in the pericycle (716 µg Se g^−1^; Fig. 7A; Supplementary Table S6; Supplementary Figs. S2 and S4). The remnant cortex and spots in the secondary xylem appeared enriched in Ca. The primary xylem contained the highest amount of Zn overall (83.8 µg Zn g^−1^). In hydroponics, *N. amplexicaulis* developed a thick taproot surrounded by a retained wall of spongy, dense cortex tissue, which was lost during histological analysis (Supplementary Fig. S2D). This cortex tissue had a high K concentration (Fig. 7, B and C; Supplementary Table S6). In taproots of hydroponically grown plants, Se was present in notable concentrations (up to 723 µg Se g^−1^) in the pericycle. Selenium was found in much lower concentrations throughout the xylem, secondary phloem, and the vascular cambium (≤210 µg Se g^−1^). Zinc was found in the vascular cambium, and in lower concentrations in the xylem and secondary phloem, and Ca was mostly found in the secondary phloem. Emerged and developing lateral roots from a hydroponics taproot showed a high Se concentration at the pericycle boundary, equivalent to, or higher than, concentrations found in the pericycle (730 to 1,490 µg Se g^−1^; Fig. 7C). Indeed, newly growing lateral roots, as seen from the surface of a developed root, had high Se (3,200 to 3,780 µg Se g^−1^) and are very low in K (Fig. 7C; Fig. 8B; Supplementary Tables S5 and S6).

Selenium in fine roots was highly present in the root tip, becoming less concentrated in the region of maturation (Fig. 8). In the elongation region, Se is concentrated in the provascular tissues (1,360 µg Se g^−1^), with some amounts appearing throughout the ground tissue (877 µg Se g^−1^; Fig. 8C; Supplementary Table S5). The overlap boundary between the elongation zone and the differentiation region exhibited a high concentration of Se in all tissues (1,170 µg Se g^−1^), before becoming depleted throughout the rest of the lateral root. This zone also contained high concentrations of Zn and Ca compared to the rest of the root tip, and lower concentrations of Zn occurred from this point onwards along the maturation zones of the fine roots (Fig. 8, A and C). The roots exhibited extensive mucigel produced from the sloughed off root cap, rich in Ca, Fe, and Mn, with bands of Se in low concentrations (Supplementary Fig. S5).

### Pulse chase experiment to determine selenium translocation

**Figure 9.**
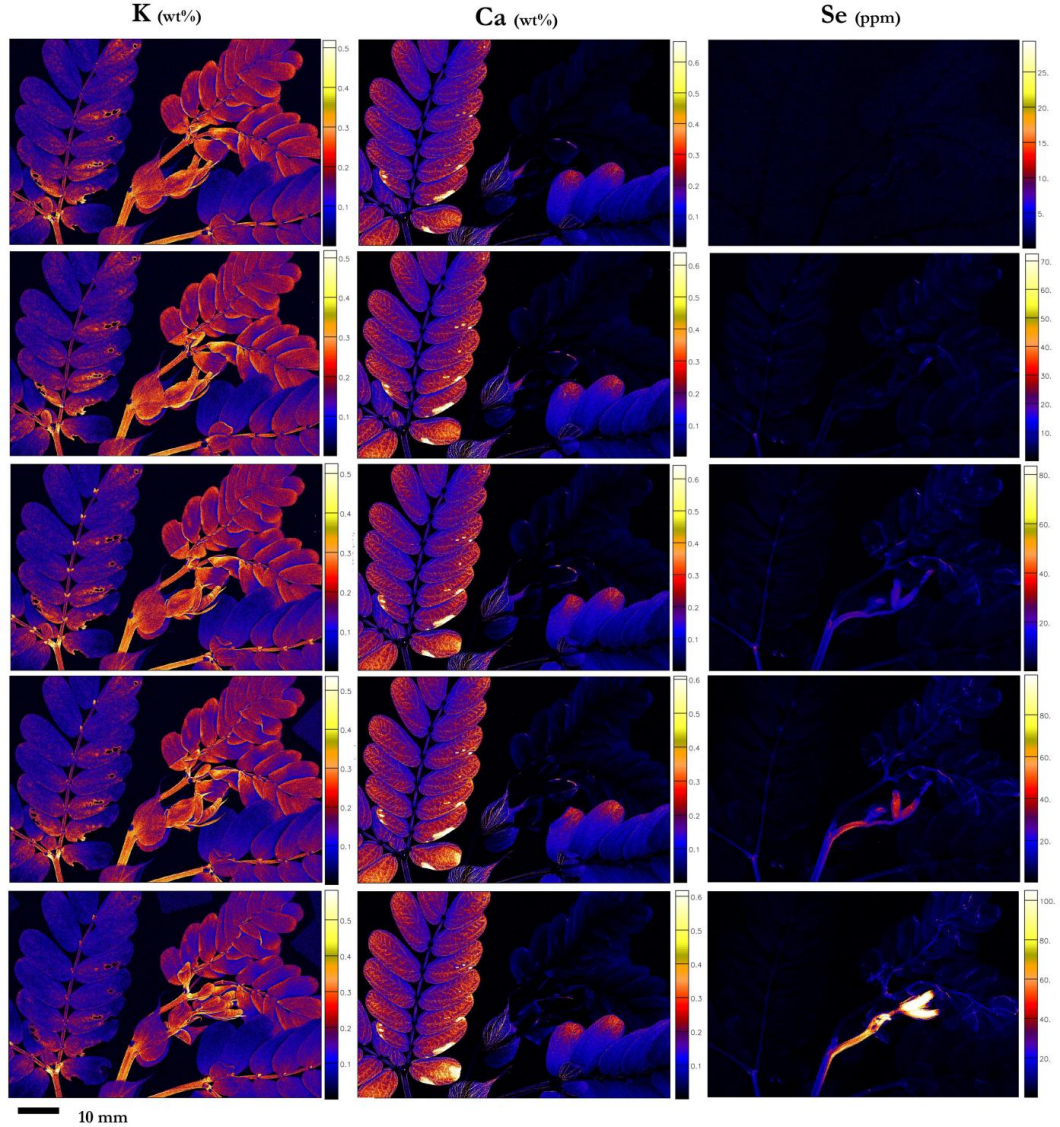
Synchrotron-based XFM elemental maps showing the distribution of K, Ca, and Se in hydrated shoot section of *N. amplexicaulis* pulse chase experiment. Plant grown in 0 Se hydroponics (r1) scanned at T0 (top row), following rows show scans at 3, 18, 50, and 74 h after the first exposure to 150 µM Se solution. Scale bar denotes 10 mm for all images. Color bars show elemental concentrations in µg g^−1^ (ppm; for Se images) or weight percent (wt%; for K and Ca images).

**Figure 10.**
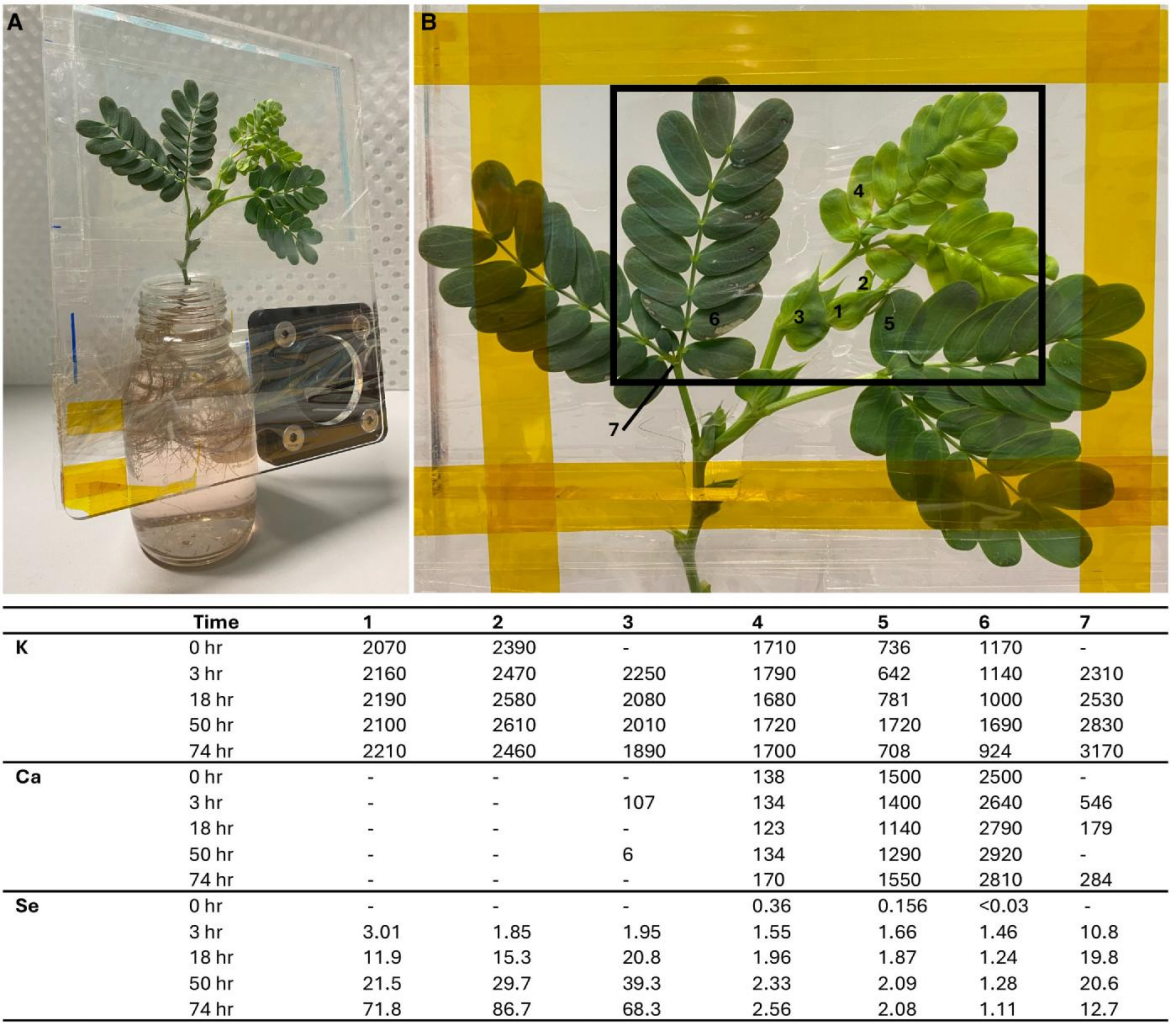
Pulse chase experiment setup and map of analyzed tissues with K, Ca, and Se concentrations (in µg g^−1^) of each analyzed tissue at each scan timepoint. **A)** sample preparation between scans, with plant mounted on stage and roots submerged in Se-dosed hydroponics solution and **B)** overview of the scan area, with tissues analyzed for average elemental concentrations using GEOPIXE labeled 1: apical meristem bundle, 2: youngest developing leaves, 3: stem leading to apical tissues, 4: basal leaflet of next youngest pinna, 5: leaflet of intermediate-age pinna, 6: leaflet of oldest pinna, 7: oldest secondary pulvinus node.

The area of view for the selected control plant exhibited an apical meristem tissue bundle, two sets of underdeveloped, unopened pinna, one set of opened “young” leaves, and two sets of old leaves (Figs. 9 and 10). The pre-exposure image showed a depletion of Se in all tissues (<1*  µ*g Se g^−1^). At 3 h of exposure to Se, a minimal presence of Se (up to 3 µg Se g^−1^) was detected in leaf tissues, with slightly more Se (10.8 µg Se g^−1^) occurring just below the secondary pulvini of the oldest set of leaves. At 18 h after Se exposure began, Se had begun to appear in concentrations up to 20.8 µg Se g^−1^ in the underdeveloped set of leaves and the youngest section of stem. At the point of 50 h of exposure, Se was present at concentrations up to 39.3 µg Se g^−1^ in the two sets of unopened leaves and the youngest section of stem. Low concentrations (2.33 µg Se g^−1^) of Se occurred in the youngest fully developed leaves, with decreasing concentrations in older leaflets, and Se was still present in the secondary pulvini of the oldest leaves (20.6 µg Se g^−1^). This pattern continued to be observed in the final image post-exposure, at 74 h. At this point, the undeveloped leaves were accumulating up to 86.7 µg Se g^−1^ and the youngest section of stem contained 68.3 µg Se g^−1^, with a higher Se concentration in the stem leading to the apical meristem bundle, as opposed to the second youngest undeveloped leaves. Accumulation of Se in the older leaflets had not exceeded 2.56 µg Se g^−1^, and the concentration in the pulvini node had decreased to 12.7 µg Se g^−1^. The patterns of K and Ca distribution remained within a similar range throughout the 74-h exposure period, except where the underdeveloped leaves had increased in size over the exposure period and Ca accumulation had increased in the damaged sections of old leaves. By the end of the experiment, the Se concentrations in organ tissues were 165 µg Se g^−1^ in young leaves/apical meristem bundle, only 17.8 µg Se g^−1^ in old leaves, 151 µg Se g^−1^ in (non-apical) stem, and 310 µg Se g^−1^ in the roots (post-experiment ICP-AES validation).

## Discussion

Through the application of synchrotron-based XFM and the pulse chase technique, we elucidated Se distribution in *N. amplexicaulis* at tissue level and posit a phloem-sink model of Se distribution in this species. Previously, Se distribution in *N. amplexicaulis* had been investigated using laboratory micro-analysis XRF and compared with other hyperaccumulator species (Harvey et al. 2020; van der Ent et al. 2023). Here, we have elucidated Se accumulation in fine root tips and shed light on the role of the pericycle in root Se transport, the movement of Se through the xylem and phloem, and the rate of Se uptake into the youngest tissues. Furthermore, we were able to closely examine how Se was distributed in the comparison species *N. heliophila*.

### The implications of selenium distribution in the root system

The roots of hydroponically grown plants showed remarkable development of the spongy cortex alongside visible retained root cap mucigel and a lack of observable root hairs on the fine root surface. These are structural developments unique to the analysis of hydroponically grown plants. Previous analyses have shown high prevailing Se concentrations in the whole root system (Harvey et al. 2020), and Se in the meristematic region of the finer, lateral roots suggests this region may play a strong role in the foraging and uptake of Se (Pinto Irish et al. 2021). The zone of elongation is likely a key area for Se uptake and transport as this tissue is highly permeable, and Se is seemingly concentrated around the provascular tissues. The unusually long root tips sometimes observed would encourage increased uptake, though this could be merely an effect of hydroponics media altering root structure. The elongation–maturation zone boundary is ubiquitously concentrated in Se and Zn, where it would have to cross the epidermis and the now-developed cortex to be transported by the proto/metaxylem once it has sufficiently developed. Development of solute barriers in this tissue may play a role in the high “micronutrient” (in-so-far as Se is not considered a nutrient) concentrations in the outer tissues at this boundary. In the mature section of the fine roots, Se would be limited in entering the vascular cylinder by the Casparian strip; this action would more than likely be mediated by the root sulfate transporters (Brown and Shrift 1982; White et al. 2007). However, there is also a possibility that a proportion of the Se in the root tips is phloem redistributed, as the root tips are a phloem sink, which may also explain the high Se concentrations in the root tip meristem. Additionally, Se occurring in high concentrations at the pericycle (the tissue from which lateral roots emerge) would give meristematic regions an early boost of Se (as observed in Figs. 7C and 8C), and be partly responsible for the high Se concentrations in the vascular cambium.

### Selenium inside stems and taproots: structural layers

The high concentration of Se in the pericycle of the taproot is indicative of the root to shoot translocation in the plants. Sulfate transporters (SULTR2;1) present in the pericycle are linked with another sulfate transporter (SULTR3;5) that transports S from the root to the shoot when *SULTR2;1* is highly expressed (Kataoka et al. 2004). As Se is highly concentrated in the pericycle, the high expression of these sulfate transporters would represent a high degree of Se uptake and translocation (Kataoka et al. 2004; Schiavon et al. 2015). SULTR2;1 is also highly expressed in the xylem parenchyma, linking with the high Se concentrations in the xylem of the roots of soil-grown plants and in the shoots. Selenium occurring in the cortex and pith of *N. amplexicaulis* stems (but not *N. heliophila*) may suggest some small degree of sequestration of this element in these tissues, which may aid in the deterrence of non-Se specific herbivores (Hanson et al. 2004; Quinn et al. 2010; Prins et al. 2019). This is likely occurring through the same mechanisms that allows sulfate and phosphate unloading from xylem fluids into nearby cells—in some cases, these compounds may be retrieved later back into the xylem (White 2012).

### Vascular transport: xylem or phloem?

Xylem transport appears to be a major component of Se movement across the plant—it was previously supposed, due to the pattern of Se accumulation in leaves and the multidirectional flow, that the phloem plays a strong role in Se transport for this species (Harvey et al. 2020). In fact, in many other species phloem transport has been assumed to be the key Se transport director, first suggested by the acknowledgment of the phloem distribution of S-analogue amino acid compounds (Bourgis et al. 1999; Hell et al. 2010). The patterns of high Se in the shoot apex, seeds and roots of this species mimics the typical sink-based phloem transport model, where the unloading of solutes (such as Se) in developing tissues drives a positive pressure flow, whereas xylem mediated transport of solutes would result in a higher deposition of solutes in the more photosynthetically active, older leaves (White 2012). However, the stem cross-sections suggest that Se is also as present in the xylem as the phloem, while being highly present in the root xylem. Comparatively, van der Ent et al. (2023) demonstrated Se to be far more present in the phloem than the xylem in freehand sections and virtual tomographic slices of roots. There could be a multitude of reasons for this discrepancy, as the samples in the latter study were all grown in soil, and roots undergoing tomographic analysis were thinner and younger than the roots in our study. Older, thicker tissues exposed to a high concentration of bioavailable Se may need to transport Se through the xylem because of physiological development and environmental Se saturation. Unfortunately, it is difficult to gather definitive conclusions about vascular transport from hand cut sections; given they were cut using a vibratome, some of the more mobile compounds trapped in larger phloem cells, metaxylem and the apoplast may have leached or smeared during the cutting process (van der Ent et al. 2018). Likely, Se in the phloem would have predominantly SeCT and could have easily leached, thus interpretations of vascular transport must be made conservatively (Peterson and Butler 1967; van der Ent et al. 2018). Regardless, mid-sections of the stem and (soil-grown) roots, as we have observed here, would still have a strong upwards flow of Se indicative of xylem transport (Mengel and Kirkby 2001), which may confer an efficiency of rapid hyperaccumulation in *N. amplexicaulis*. The Se present in the xylem may result in small amounts of Se being transferred to older leaves, as observed in the pulse chase experiment, and may also contribute to the universally high concentrations across undeveloped young leaf tissues. Protoxylem transporting solutes to the youngest apical tissues are frequently destroyed in rapidly elongating and expanding shoot growth (Esau 1967), which could lead to distortion of vasculature and seemingly organ-wide distribution of Se in these youngest tissues. Rapid transfer of solutes between xylem and phloem is known to occur in the leaf blade—the effect of which can be easily seen in maturing leaves, where the Se appears to be translocated through the vascular tissues (White 2012; Harvey et al. 2020).

While considering observed distribution of Se in the mature xylem, the pulse chase experiment draws attention to the role of the secondary pulvinus in directing distribution of Se in older pinna. As Se is being transported toward older pinna through the xylem, we can observe very low Se concentrations in the older pinna, but a high yet fluctuating Se concentration in the secondary pulvini. This visualization is often confounded in already Se-exposed plants, as the older leaflets would still be participating in multidirectional Se flow. A link between Se directionality and pulvini in this species has been made before, and the role of the pulvinus in directing xylem resistance and solute transfer has been known for some time (Neumann 1987; Harvey et al. 2020). In *Mimosa pudica*, a species famous for its highly responsive pulvini, the secondary pulvinus allows the pinna to bend down and away in a touch response; an action often attributed to turgidity of motor cells and ion fluxes (Abe 1981; Hill and Findlay 1981). This organ has been imaged with several independent lignified xylem bundles, bending flexibly (Song et al. 2014). Having multiple xylem bundles allows for a greater xylem surface area in the pulvinus and subsequently a higher hydraulic conductance, and thus greater opportunity for exchange between cells. The tertiary pulvini of *N. amplexicaulis* can affect the same mechanical response, though are slower to react, and the secondary pulvinus is capable of this action as well, if only through diurnal cycles and less so through touch stimulus (Windler 1966; personal observation). If high xylem surface areas and changes in lignification due to flexibility are also present in this organ, it could be the ideal location to shift Se from xylem to phloem cells, bypassing the pinna themselves (White 2012). Further investigation of the morphology of this organ may be warranted when studying Se hyperaccumulation in *N. amplexicaulis*.

An additional conclusion about the pulse chase experiment was the similarly high accumulation of Se in not just the youngest set of developing pinna, but in *all* sets of the young, developing pinna. This reaffirms the role of phloem in Se distribution and leads to our main hypothesis: Se is transported through the xylem and phloem to the leaves and developing tissue and is transferred to the phloem in the leaf blades once sink becomes source, or redirected in the pulvini. Phloem sinks in the youngest developing leaves and tissues concentrate Se into these tissues specifically. Similarly, the concept of xylem uptake and phloem redistribution has been a proposed model for other studied hyperaccumulators (Pilon-Smits 2019). Remobilization of Se is undoubtedly highly important in this species, when considering the potential for phloem redistribution into the root tips as well. Plant growth increases substantially upon exposure to Se, with little change in biomass between lower and higher doses of Se; phloem redistribution is likely playing a role in the lack of significant differences in size between low and high doses. The effects of insufficient Se remobilization can result in smaller plants, or malformed young leaves after enough time (M-A. Harvey, unpublished data). The phloem-sink driven distribution and the role of the pulvinus in redirecting flow may be verified using ^75^Se isotope tracing approaches (Wiggenhauser et al. 2022). Many of the earlier investigations on *N. amplexicaulis* used this methodology to understand the accumulation capacity under exposure, and literature has many examples of this isotope being successfully applied to other species (Peterson and Butler 1962; Peterson and Butler 1967; Martin et al. 1971; Goodson et al. 2003).

### Toxicity of selenium in *N. heliophila*

The *N. heliophila* stem and leaf organs had a very similar Se distribution compared to *N. amplexicaulis*, and arguably, similar processes of Se uptake and distribution are at play here. Selenium accumulation occurs in the same tissues with a similar distributional pattern, though with a less distinct capacity for retention in specific tissues or concentration in specific organs—an example of this concept can be seen in the leaves—the rachilla of young *N. amplexicaulis* leaves are often very low in Se, where Se is inside the leaflet vascular tissue (Harvey et al. 2020). In *N. heliophila*, the rachilla and the leaflet vasculature areas contain similar levels of Se concentration, akin to observations of slightly older leaflets of *N. amplexicaulis* where the Se has largely left the pinna (van der Ent et al. 2023). Likewise, *S. pinnata* has been measured with Se primarily concentrated at the epidermal cells and leaf margins, with a similar but less distinctive and controlled accumulation occurring at the margins of *Stanelya albescens*, a non-accumulator plant (Freeman et al. 2010). This mirrors the pattern observed at a whole plant scale between *N. amplexicaulis* and *N. heliophila*, if only with the Se concentrated in the vascular structures instead (Harvey et al. 2020). Likely these similarities can be drawn on the basis of evolutionary relationship and morphological resemblance. Given that Se in *N. heliophila* has been observed with a proportion of organic Se (J.K. Kirby, unpublished data), a phloem-sink model of Se remobilization to the young leaves is also possible for this species (Pickering et al. 2003).

While low doses of Se showed little difference in biomass compared to the control, *N. heliophila* dosed with selenate had higher Se accumulation and a variety of damage to its leaves, some were malformed and shriveled, others were necrotic and dried at the tips. Interestingly, these two types of damages resulted in different Se patterns—the malformed leaves had less vascular Se distribution in the damaged areas, whereas necrotic sections of leaves were highly concentrated in Se. *N. heliophila* probably unloads Se within the dead tissues as tolerance mechanism—this has been observed in other hyperaccumulators and metallophytes where “toxic” elements are pumped into damaged tissues, or leaf senescence is induced (Dahmani-Muller et al. 2000). Selenite exposure, however, elicited lower toxicity effects and lower Se accumulation in this species and selenite exposed plants, particularly at higher concentrations, suffered less reduction in biomass compared to selenate exposed plants. Still, the capacity for this species to uptake and survive Se exposure, up to 25 µM Se, is interesting—potentially an adaptation to growing on seleniferous soils alongside and often right next to *N. amplexicaulis* (Harvey et al. 2024).

## Conclusions

From observations of the distribution of Se in *N. amplexicaulis* and *N. heliophila*, we hypothesize that Se distribution is driven by a phloem-sink-based model, though Se is present in both phloem and xylem tissues. The high enrichment if Se in the root tips may be both indicative of this model and the key entry site for Se transport. Future research should work to determine the role of the pulvini in Se distribution, detail the Se distribution in the meristem tissues, and advance the understanding of the mechanistic drivers of this phloem-sink model.

## Materials and methods

### Seed collection and culture conditions

Seeds of *N. amplexicaulis* and *N. heliophila* were sourced from plants growing in the native habitat at Silver Hills Station near Richmond, Queensland, Australia (−20.648359, 143.098375). Seeds were germinated by piercing with a scalpel and leaving to soak in water for 24 h before being transferred to shallow containers filled with moistened 50% fine perlite and 50% vermiculite. The seeds were left for 1 wk, and germinated specimens with fully emerged cotyledons and first adult leaves were transferred into hydroponics culture. The hydroponic system consisted of separate rectangular containers (11 cm height × 30 cm width × 40 cm length; 11 L each with 8 foam baskets for individual plants) filled with Aqua Vega Classic nutrient solution (Canna, Brisbane QLD, Australia) diluted as 10 mL each of A and B solutions in 11L DI water (∼half strength), with an additional 3 mL of 20 mM FeHBED (iron N,N′-bis (2-hydroxybenzyl)ethylenediamine-N,N′-diacetic acid) solution. The final diluted solution contained: 1.31 mM N, 0.51 mM P, 2.88 mM K, 0.43 mM Ca, 0.34 mM Mg, 0.31 mM S, 0.61 μM Fe-DTPA, 0.44 μM Fe-EDDHA, 5.45 μM FeHBED, 10 μM B, 0.14 μM Cu, 3.3 μM Mn, 0.2 μM Mn-DTPA, 0.28 μM Mo, and 1.53 μM Zn. The solution was kept constantly aerated using a 25 cm long air stone at the bottom of each container. The nutrient solution was spiked with Se treatment as the Na_2_SeO_4_ (for selenate) or Na_2_SeO_3_ (for selenite) salt 0.1 M Se stock solution to obtain desired Se concentrations. The nutrient solution pH was maintained at pH buffer range of 5.8–6.1 by additions of 0.1 M KOH or 0.1 M HNO_3_ solution as required, and solutions were changed completely once a week. In each treatment, 8 replicate plants were grown in 3 cm round plastic baskets with a foam disk to allow the roots to be immersed in the nutrient solution. Plants were grown for 60 d with a 12–12 h light–dark photoperiod, under high intensity LED lights VYPRx PLUS with PhysioSpec Indoor spectra (Fluence Bioengineering, Austin, TX, USA), with a photosynthetic photon flux density of 550 μmol m^2^ s^−1^ (measured with an Apogee MQ-500 instrument) at 22/20 °C day/night. *Synchrotron analysis*: Selenium salts were added to achieve nominal Se concentrations of 75 μM Se total for *N. amplexicaulis*, and 25 μM Se total for *N. heliophila.* Treatments for *N. amplexicaulis* included (i) control (0 Se), (ii) 75 μM selenate, (iii) 75 μM selenite, (iv) 25:50 μM selenate:selenite, and (v) 50:25 μM selenate:selenite. Treatments for *N. heliophila* included (i) 25 μM selenate and (ii) 25 μM selenite. Plants were grown for 5.5 wk then transported to the Synchrotron for analysis. Identical hydroponics systems and methodology was utilized at the Australian Synchrotron in order to keep the plants alive and fresh up until the moment of sectioning for analysis. *Dosing trial*: Selenium salts were added to achieve 0 (control), 25, 50, and 100 μM Se using either selenate or selenite per treatment for *N. amplexicaulis*. Additionally, doses of 0, 10, 25, and 50 μM Se using either selenate or selenite per treatment were added for *N. heliophila*. Plants were grown for 6 wks, before 6 plants per treatment were selected for harvest.

### Synchrotron-based X-ray Fluorescence Microscopy

The XFM beamline at the Australian Synchrotron employs a Si(111) monochromator and a pair of Kirkpatrick–Baez mirrors to deliver X-rays onto the specimen with fluorescent X-rays collected in a backscatter geometry using the 384-element Maia detector system (Lombi et al. 2011; Paterson et al. 2011; Howard et al. 2020). The Maia detector uses a large detector array to maximize detected signal and count-rates for efficient imaging, and an annular detector geometry, where the beam passes through the detector and strikes the sample at normal incidence (Kirkham et al. 2010; Siddons et al. 2014). This enables a large solid angle (1.2 steradians) to be achieved to either maximize efficiency or to reduce the dose and potential damage to a specimen (Ryan et al. 2010, 2018). The possibility of radiation-induced damage in synchrotron-based XFM analysis is an important consideration; in hydrated plant tissue, assessed dose-limits are 4.1 kGy before detectable damage occurs (van der Ent et al. 2018; Jones et al. 2019). Fast scanning (per-pixel dwell time is less than 10 ms) was used to limit the radiation dose and thus damage. The samples were held between 2 sheets of Ultralene thin film (4.0 μm) stretched over a Perspex frame to prevent dehydration during the measurement. For the 2D elemental mapping of whole small plants and tissue (*i.e.* roots, stem, and leaf cross-sections) were analyzed. The tissue samples for roots and stems were embedded in agarose then sectioned with a vibratome at 40 μm thickness using a “dry knife” method (*i.e.* without PBS solution to avoid elemental deportment and losses), then mounted between two sheets of 4.0 μm Ultralene thin film. For leaf samples, soil-grown taproot samples, and *N. heliophila* stems samples “dry knife” hand cutting using a fresh razor blade and a plastic block to cut against was employed. Low resolution “overview scans” of multiple tissues or sections of tissue were taken, with detailed scans of the best-prepared samples following. Where differences in elemental distribution were observed, multiple detailed scans were collected. In the “pulse chase” experiment, plants from the Se-control treatment were used and following the methodology of Blamey et al. (2018) a single plant was mounted on a scanning frame backed with tape and covered with film with roots secured to frame in a moistened plastic package. The whole shoot was used for the synchrotron-based XFM analysis, then the roots were submerged in a glass bottle with nutrient solution to which Se stock solution was added to reach 150 μM Se with equal parts selenate:selenite. The thin film was removed from the leaf surfaces to allow for transpiration. The shoot was scanned again 3 h after exposure to Se began, and further scans were acquired at 18, 50, and 74 h, where roots were exposed to fresh Se solution and plant was able to transpire in between scans.

#### Chemical analysis of dosing experiment samples

The samples from the hydroponics dosing experiments, and samples from the pulse chase specimen post-analysis, were separated into young leaves, old leaves, stem and taproot and fine roots and dried at 60 °C for 72 h. The separated parts were weighed then ground to a fine powder in an impact mill at 19,000 rpm (IKA Tube Mill 100 control with disposable titanium blades) and weighed to 100 ± 5 mg in 6 mL polypropylene tubes. Samples < 100 mg were weighed directly into the tubes. These samples were predigested using 2 mL HNO_3_ (70%) for 24 h before being digested in a block heater (Thermo Scientific digital dry bath) for a 2-h program (1 h at 70 °C followed by 1 h at 125 °C) and diluted to 10 mL with ultrapure water (Millipore 18.2 MΩ·cm at 25 °C) before analysis with Inductively coupled plasma atomic emission spectroscopy (ICP-AES) using a Thermo Scientific iCAP 7400 instrument. All elements were calibrated with a 4-point curve covering analyte ranges in the samples. In-line internal addition standardization using yttrium was used to compensate for matrix-effect interferences. Quality controls included matrix blanks, Standard Reference Material (NIST Apple Leaves 1515), and our own internal reference materials (homogenized powdered *N. amplexicaulis* tissues).

### Plant tissue histology

Hydroponically and soil cultivated specimens of *N. amplexicaulis* and *N. heliophila* were prepared for histological analysis (Supplementary Fig. S2). Leaf, stem, and taproot sections of *N. amplexicaulis* and leaf and stem sections of *N. heliophila* were dissected into 0.5 cm lengths. The dissected samples were fixed with FAA solution (5% formaldehyde, 10% acetic acid and 50% ethanol) for 24 h. The tissue samples were dehydrated in ethanol, processed in a Leica ASP300S processor with ethanol and xylene, and embedded in paraffin wax through a Leica HistoCore Arcadia H embedding station. The tissue samples were sectioned transversely at 5 *µ*m using a Leica RM2245 rotary microtome and placed on Super frost Plus slides. The histological sections were stained with 1% sodium acetate buffered solution and 0.1% Toluidine blue in 1% sodium acetate buffer solution. The tissue sections were cleared in xylene 3 times, mounted on slides using DepeX and covered with coverslips. The tissue sample sections were observed and imaged on a Zeiss AxioScan Z1 with a Plan Apochromat 20× objective and Hitachi HV-F203SCL camera (with 200 µs exposure and extended depth of focus).

### Data analysis

The incident energy used for the whole XFM experiment was 15.8 keV and the XRF event stream was analyzed using the Dynamic Analysis method as implemented in GeoPIXE (Ryan et al. 1990, 2005; Ryan and Jamieson 1993; Ryan 2000). This method generates elemental images, which are (i) overlap-resolved, (ii) with subtracted background, and (iii) quantitative, *i.e.* in μg g^−1^ dry weight units. The matrix used for modeling was a cellulose-hydrate (as an approximation of hydrated plant material with the empirical formula of C_12_H_24_O_12_) with a density of 1.2 g cm^3^ and a thickness of 300 μm. GeoPIXE was additionally used to manually outline plant tissue regions and perform analysis of average elemental concentrations for each tissue. Statistical analysis of dosing trial samples was conducted using R version 4.3.2 and RStudio version 2024.09.1+394 (Integrated Development for R. RStudio, PBC, Boston, MA, http://www.rstudio.com) and Microsoft Excel 2016 (Redmond). Boxplot was produced using packages ggplot2, gridExtra, and viridis. Statistical tests performed using package rstatix. Shapiro test and Levene's test were used to determine which treatments could be analyzed with ANOVA or Kruskal–Wallis test (Supplementary Table S1).

## Supplementary Material

kiaf367_Supplementary_Data

## Data Availability

All data used and generated during the writing of this manuscript may be accessed upon reasonable request to the corresponding author.

## Supplementary data

The following materials are available in the online version of this article.

**Supplementary Figure S1.** Photographs and light microscope images of plant tissues used in synchrotron XFM elemental maps.

**Supplementary Figure S2.** Light micrographs showing Toluidine blue-stained tissue cross-sections of *Neptunia amplexicaulis* and *Neptunia heliophila*.

**Supplementary Figure S3.** Synchrotron XFM elemental maps showing the distribution of K, Ca, and Se in hydrated leaf sections of *Neptunia heliophila*.

**Supplementary Figure S4.** Light microscope images of *Neptunia amplexicaulis* plant tissues used in synchrotron XFM elemental maps.

**Supplementary Figure S5.** Synchrotron XFM elemental maps showing the distribution of K, Ca, Fe, Mn, Zn, and Se in hydrated fine roots of *N. amplexicaulis* with prominent encasing mucigel.

**Supplementary Table S1.** Results of statistical tests run on selenium concentration by plant tissue data, subset into treatment combination, and on whole plant dry weight (dw) biomass data, subset into species.

**Supplementary Table S2.** Results of post hoc tests run on selenium concentration by plant tissue data, subset into treatment combination, and whole plant biomass by treatment combination.

**Supplementary Table S3.** The whole plant biomass minimum, maximum range, and mean in for each treatment.

**Supplementary Table S4.** The Se concentration minimum, maximum range, and mean in each plant tissue for each treatment.

**Supplementary Table S5.** Average elemental concentrations (S, K, Ca, Zn, and Se) of tissues from the synchrotron XFM elemental maps of whole plant areas from Figs. 4, 5, and 8.

**Supplementary Table S6.** Average elemental concentrations (K, Ca, Zn, and Se) of tissues from the synchrotron XFM elemental maps of cross-sections from Figs. 4, 6, and 7.

## Dive Curated Terms

The following phenotypic, genotypic, and functional terms are of significance to the work described in this paper:

selenium CHEBI: CHEBI:27568

zinc CHEBI: CHEBI:27363

calcium CHEBI: CHEBI:29320

potassium CHEBI: CHEBI:85264

whole plant AmiGo: PO:0000003

## References

[kiaf367-B1] Abe T . Chloride ion efflux during an action potential in the main pulvinus of *Mimosa pudica*. J Plant Res. 1981:94(4):379–383. 10.1007/BF02493398

[kiaf367-B2] Anderson JW . Selenium interactions in sulfur metabolism. Sulfur nutrition and assimilation in higher plants: regulatory, agricultural and environmental aspects. The Hague, The Netherlands: SPB Academic Publishing; 1993. p. 49–60.

[kiaf367-B70] Atlas of Living Australia. [accessed 2022 Jun 26]. https://biocache.ala.org.au/occurrences/search?q=lsid%3Ahttps%3A%2F%2Fid.biodiversity.org.au%2Fnode%2Fapni%2F2898267&qualityProfile=ALA.

[kiaf367-B3] Bean AR . A revision of *Neptunia* Lour. (Leguminosae: subfamily Caesalpinioideae, Mimosoid clade) in Australia and Malesia. Austrobaileya. 2022:12:59–106. 10.5962/p.366317

[kiaf367-B4] Beath OA, Draize JH, Eppson HF, Gilbert CS, McCreary OC. Certain poisonous plants of Wyoming activated by selenium, and their association with respect to soil types. J Am Pharm Assoc. 1934:23:94–97. 10.1002/jps.3080230204

[kiaf367-B5] Beath OA, Gilbert CS, Eppson HF. The use of indicator plants in locating seleniferous areas in western United States. I. General. Am J Bot. 1939:26(4):257–269. 10.1002/j.1537-2197.1939.tb12900.x

[kiaf367-B6] Blamey FPC, Paterson DJ, Walsh A, Afshar N, McKenna BA, Cheng M, Tang C, Horst WJ, Menzies NW, Kopittke PM. Time-resolved X-ray fluorescence analysis of element distribution and concentration in living plants: an example using manganese toxicity in cowpea leaves. Environ Exp Bot. 2018:156:151–160. 10.1016/j.envexpbot.2018.09.002

[kiaf367-B7] Both EB, Stonehouse GC, Lima LW, Fakra SC, Aguirre B, Wangeline AL, Xiang J, Yin H, Jókai Z, Soós Á, et al Selenium tolerance, accumulation, localization and speciation in a *Cardamine* hyperaccumulator and a non-hyperaccumulator. Sci Total Environ. 2020:703:135041. 10.1016/j.scitotenv.2019.135041 31767332 PMC7060786

[kiaf367-B8] Bourgis F, Roje S, Nuccio ML, Fisher DB, Tarczynski MC, Li C, Herschbach C, Rennenberg H, Pimenta MJ, Shen T-L, et al S-methylmethionine plays a major role in phloem sulfur transport and is synthesized by a novel type of methyltransferase. Plant Cell. 1999:11(8):1485–1497. 10.1105/tpc.11.8.1485 10449582 PMC144290

[kiaf367-B9] Brown T, Shrift A. Selenium: toxicity and tolerance in higher plants. Biol Rev Camb Philos Soc. 1982:57(1):59–84. 10.1111/j.1469-185X.1982.tb00364.x

[kiaf367-B10] Cappa J, Pilon-Smits E. Evolutionary aspects of elemental hyperaccumulation. Planta. 2014:239(2):267–275. 10.1007/s00425-013-1983-0 24463931

[kiaf367-B11] Dahmani-Muller H, van Oort F, Gélie B, Balabane M. Strategies of heavy metal uptake by three plant species growing near a metal smelter. Environmental Pollution. 2000:109(2):231–238. 10.1016/S0269-7491(99)00262-6 15092894

[kiaf367-B12] Esau K . Plant anatomy. United States of America: John Wiley & Sons, Inc; 1967.

[kiaf367-B13] Freeman J, Tamaoki M, Stushnoff C, Quinn CF, Cappa JJ, Devonshire J, Fakra S, Marcus M, McGrath SP, Van Hoewyk D, et al Molecular mechanisms of selenium tolerance and hyperaccumulation in *Stanleya pinnata*. Plant Physiol. 2010:153(4):1630–1652. 10.1104/pp.110.156570 20498337 PMC2923907

[kiaf367-B14] Freeman JL, Zhang LH, Marcus MA, Fakra S, McGrath SP, Pilon-Smits EAH. Spatial imaging, speciation, and quantification of selenium in the hyperaccumulator plants *Astragalus bisulcatus* and *Stanleya pinnata*. Plant Physiol. 2006:142(1):124–134. 10.1104/pp.106.081158 16920881 PMC1557614

[kiaf367-B15] Goodson CC, Parker DR, Amrhein C, Zhang Y. Soil selenium uptake and root system development in plant taxa differing in Se-accumulating capability. New Phytol. 2003:159(2):391–401. 10.1046/j.1469-8137.2003.00781.x 33873369

[kiaf367-B16] Hanson B, Lindblom SD, Loeffler ML, Pilon-Smits EA. Selenium protects plants from phloem-feeding aphids due to both deterrence and toxicity. New Phytol. 2004:162(3):655–662. 10.1111/j.1469-8137.2004.01067.x 33873760

[kiaf367-B17] Harvey M-A, Erskine PD, Harris HH, Brown GK, Pilon-Smits EAH, Casey LW, Echevarria G, van Der Ent A. Distribution and chemical form of selenium in *Neptunia amplexicaulis* from Central Queensland, Australia. Metallomics. 2020:12(4):514–527. 10.1039/c9mt00244h 32055807

[kiaf367-B18] Harvey M-A, Erskine PD, Harris HH, Virtue JI, van Der Ent A. Plant-soil relations of selenium, molybdenum and vanadium in the Richmond District of Central Queensland, Australia. Plant Soil. 2024:504(1–2):435–445. 10.1007/s11104-024-06633-7

[kiaf367-B19] Hell R, Khan MS, Wirtz M. Cellular biology of sulfur and its functions in plants. In: Cell biology of metals and nutrients. Heidelberg (Germany): Springer; 2010. p. 243–279.

[kiaf367-B20] Hill BS, Findlay GP. The power of movement in plants: the role of osmotic machines. Q Rev Biophys. 1981:14(2):173–222. 10.1017/S0033583500002249 7025083

[kiaf367-B21] Howard DL, de Jonge MD, Afshar N, Ryan CG, Kirkham R, Reinhardt J, Kewish CM, McKinlay J, Walsh A, Divitcos J, et al The XFM beamline at the Australian Synchrotron. J Synchrotron Radiat. 2020:27(5):1447–1458. 10.1107/S1600577520010152 32876622

[kiaf367-B22] Jones MWM, Kopittke PM, Casey L, Reinhardt J, Blamey FPC, van der Ent A. Assessing radiation dose limits for X-ray fluorescence microscopy analysis of plant specimens. Ann Bot. 2019:125(4):599–610. 10.1093/aob/mcz195 PMC7102987 31777920

[kiaf367-B23] Kataoka T, Hayashi N, Yamaya T, Takahashi H. Root-to-shoot transport of sulfate in *Arabidopsis.* Evidence for the role of SULTR3;5 as a component of low-affinity sulfate transport system in the root vasculature. Plant Physiol. 2004:136(4):4198–4204. 10.1104/pp.104.045625 15531709 PMC535849

[kiaf367-B24] Kirkham R, Dunn PA, Kuczewski AJ, Siddons DP, Dodanwela R, Moorhead GF, Ryan CG, De Geronimo G, Beuttenmuller R, Pinelli D, et al The Maia spectroscopy detector system: engineering for integrated pulse capture, low-latency scanning and real-time processing. AIP Conf Proc. 2010:1234(1):240–243. 10.1063/1.3463181

[kiaf367-B25] Knott SG, McCray CWR. Two naturally occurring outbreaks of selenosis in Queensland. Aust Vet J. 1959:35(7):332–334. 10.1111/j.1751-0813.1959.tb08505.x

[kiaf367-B26] Lima LW, Pilon-Smits EAH, Schiavon M. Mechanisms of selenium hyperaccumulation in plants: a survey of molecular, biochemical and ecological cues. Biochim Biophys Acta Gen Subj. 2018:1862(11):2343–2353. 10.1016/j.bbagen.2018.03.028 29626605

[kiaf367-B27] Lombi E, de Jonge M, Donner E, Ryan C, Paterson D. Trends in hard X-ray fluorescence mapping: environmental applications in the age of fast detectors. Anal Bioanal Chem. 2011:400(6):1637 1644. 10.1007/s00216-011-4829-2 21390564

[kiaf367-B28] Martin JL, Shrift A, Gerlach ML. Use of ^75^Se-selenite for the study of selenium metabolism in *Astragalus*. Phytochemistry. 1971:10(5):945–952. 10.1016/S0031-9422(00)89922-7

[kiaf367-B29] McCray CWR, Hurwood IS. Selenosis in north west Queensland associated with marine cretaceous formation. Qld J Agric Sci. 1963:20:475–498. https://era.dpi.qld.gov.au/id/eprint/13930/

[kiaf367-B30] Mengel K, Kirkby EA. Principles of plant nutrition. Dordrecht (the Netherlands): Springer Science & Business Media; 2001.

[kiaf367-B31] Neumann PM . Sequential leaf senescence and correlatively controlled increases in xylem flow resistance. Plant Physiol. 1987:83(4):941–944. 10.1104/pp.83.4.941 16665368 PMC1056479

[kiaf367-B32] Oldfield JE . Selenium world atlas: updated edition. Grimbergen, Belgium: Selenium-Tellurium Development Association; 2002.

[kiaf367-B33] Paterson D, De Jonge M, Howard D, Lewis W, McKinlay J, Starritt A, Kusel M, Ryan C, Kirkham R, Moorhead G. The X-ray fluorescence microscopy beamline at the Australian Synchrotron. AIP Conf Proc. 2011:1365(1):219–222. 10.1063/1.3625343

[kiaf367-B34] Peterson PJ, Butler GW. The uptake and assimilation of selenite by higher plants. Aust J Biol Sci. 1962:15(1):126–146. 10.1071/BI9620126

[kiaf367-B35] Peterson PJ, Butler GW. Significance of selenocystathionine in an Australian selenium-accumulating plant, *Neptunia amplexicaulis*. Nature. 1967:213(5076):599–600. 10.1038/213599a0

[kiaf367-B36] Pickering IJ, Wright C, Bubner B, Ellis D, Persans MW, Yu EY, George GN, Prince RC, Salt DE. Chemical form and distribution of selenium and sulfur in the selenium hyperaccumulator *Astragalus bisulcatus*. Plant Physiol. 2003:131(3):1460–1467. 10.1104/pp.014787 12644695 PMC166905

[kiaf367-B37] Pilon-Smits EA . On the ecology of selenium accumulation in plants. Plants. 2019:8(7):197. 10.3390/plants8070197 31262007 PMC6681216

[kiaf367-B38] Pinto Irish K, Harvey M-A, Erskine PD, van der Ent A. Root foraging and selenium uptake in the Australian hyperaccumulator *Neptunia amplexicaulis* and non-accumulator *Neptunia gracilis*. Plant Soil. 2021:462(1–2):219–233. 10.1007/s11104-021-04843-x

[kiaf367-B39] Prins NC, Hantzis JL, Valdez-Barillas RJ, Cappa JJ, Fakra CS, Milano de Tomasel C, Wall HD, Pilon-Smits AHE. Getting to the root of selenium hyperaccumulation—localization and speciation of root selenium and its effects on nematodes. Soil Syst. 2019:3(3):47. 10.3390/soilsystems3030047

[kiaf367-B40] Quinn CF, Freeman JL, Reynolds RJB, Cappa JJ, Fakra SC, Marcus MA, Lindblom SD, Quinn EK, Bennett LE, Pilon-Smits EAH. Selenium hyperaccumulation offers protection from cell disruptor herbivores. BMC Ecol. 2010:10(1):19. 10.1186/1472-6785-10-19 20799959 PMC2940900

[kiaf367-B41] Quinn CF, Prins CN, Freeman JL, Gross AM, Hantzis LJ, Reynolds RJB, In Yang S, Covey PA, Bañuelos GS, Pickering IJ, et al Selenium accumulation in flowers and its effects on pollination. New Phytol. 2011:192(3):727–737. 10.1111/j.1469-8137.2011.03832.x 21793829

[kiaf367-B42] Rosenfeld I, Beath OA. Selenium: geobotany, biochemistry, toxicity and nutrition. New York: Academic Press; 1964.

[kiaf367-B43] Ryan C, Etschmann B, Vogt S, Maser J, Harland C, Van Achterbergh E, Legnini D. Nuclear microprobe–synchrotron synergy: towards integrated quantitative real-time elemental imaging using PIXE and SXRF. Nucl Instrum Methods Phys Res B. 2005:231(1–4):183–188. 10.1016/j.nimb.2005.01.054

[kiaf367-B44] Ryan CG . Quantitative trace element imaging using PIXE and the nuclear microprobe. Int J Imaging Syst Technol. 2000:11(4):219–230. 10.1002/ima.1007

[kiaf367-B45] Ryan CG, Cousens DR, Sie SH, Griffin WL. Quantitative analysis of PIXE spectra in geoscience applications. Nucl Instrum Methods Phys Res B. 1990:49(1–4):271–276. 10.1016/0168-583X(90)90259-W

[kiaf367-B46] Ryan CG, Jamieson DN. Dynamic analysis: on-line quantitative PIXE microanalysis and its use in overlap-resolved elemental mapping. Nucl Instrum Methods Phys Res B. 1993:77(1–4):203–214. 10.1016/0168-583X(93)95545-G

[kiaf367-B47] Ryan CG, Kirkham R, de Jonge MD, Siddons DP, van der Ent A, Pagés A, Boesenberg U, Kuczewski AJ, Dunn P, Jensen M, et al The Maia detector and event mode. Synchrotron Radiat News. 2018:31(6):21–27. 10.1080/08940886.2018.1528430

[kiaf367-B48] Ryan CG, Kirkham R, Hough RM, Moorhead G, Siddons DP, de Jonge MD, Paterson DJ, De Geronimo G, Howard DL, Cleverley JS. Elemental X-ray imaging using the Maia detector array: the benefits and challenges of large solid-angle. Nucl Instrum Methods Phys Res B. 2010:619(1–3):37–43. 10.1016/j.nima.2009.11.035

[kiaf367-B49] Schiavon M, Pilon M, Malagoli M, Pilon-Smits EAH. Exploring the importance of sulfate transporters and ATP sulphurylases for selenium hyperaccumulation—a comparison of *Stanleya pinnata* and *Brassica juncea* (Brassicaceae). Front Plant Sci. 2015:6:2. 10.3389/fpls.2015.00002 25688247 PMC4304243

[kiaf367-B50] Schiavon M, Pilon-Smits EA. The fascinating facets of plant selenium accumulation—biochemistry, physiology, evolution and ecology. New Phytol. 2017:213(4):1582–1596. 10.1111/nph.14378 27991670

[kiaf367-B51] Siddons D, Kirkham R, Ryan C, De Geronimo G, Dragone A, Kuczewski A, Li Z, Carini G, Pinelli D, Beuttenmuller R. Maia X-ray microprobe detector array system. J Phys. 2014:499:012001. 10.1088/1742-6596/499/1/012001

[kiaf367-B52] Song K, Yeom E, Lee SJ. Real-time imaging of pulvinus bending in *Mimosa pudica*. Sci Rep. 2014:4(1):6466. 10.1038/srep06466 25253083 PMC5377328

[kiaf367-B53] van der Ent A, Przybyłowicz WJ, de Jonge MD, Harris HH, Ryan CG, Tylko G, Paterson DJ, Barnabas AD, Kopittke PM, Mesjasz-Przybyłowicz J. X-ray elemental mapping techniques for elucidating the ecophysiology of hyperaccumulator plants. New Phytol. 2018:218(2):432–452. 10.1111/nph.14810 28994153

[kiaf367-B54] van der Ent A, Salinitro M, Brueckner D, Spiers KM, Montanari S, Tassoni A, Schiavon M. Differences and similarities in selenium biopathways in *Astragalus, Neptunia* (Fabaceae) and *Stanleya* (Brassicaceae) hyperaccumulators. Ann Bot. 2023:132(2):349–361. 10.1093/aob/mcad110 37602676 PMC10583200

[kiaf367-B55] White PJ . Chapter 3—long-distance transport in the xylem and phloem. In: Marschner P, editor. Marschner's mineral nutrition of higher plants. 3rd ed. San Diego: Academic Press; 2012. p. 49–70.

[kiaf367-B56] White PJ . Selenium accumulation by plants. Ann Bot. 2016:117(2):217–235. 10.1093/aob/mcv180 26718221 PMC4724052

[kiaf367-B57] White PJ, Bowen HC, Marshall B, Broadley MR. Extraordinarily high leaf selenium to sulfur ratios define ‘Se-accumulator’ plants. Ann Bot. 2007:100(1):111–118. 10.1093/aob/mcm084 17525099 PMC2735298

[kiaf367-B58] White PJ, Bowen HC, Parmaguru P, Fritz M, Spracklen WP, Spiby RE, Meacham MC, Mead A, Harriman M, Trueman LJ, et al Interactions between selenium and sulphur nutrition in *Arabidopsis thaliana*. J Exp Bot. 2004:55(404):1927–1937. 10.1093/jxb/erh192 15258164

[kiaf367-B59] Wiggenhauser M, Moore R, Wang P, Bienert GP, Laursen KH, Blotevogel S. Stable isotope fractionation of metals and metalloids in plants: a review. Front Plant Sci. 2022:13:840941. 10.3389/fpls.2022.840941 35519812 PMC9063737

[kiaf367-B60] Windler DR . A revision of the genus *Neptunia* (Leguminosae). Aust J Bot. 1966:14(3):379–420. 10.1071/BT9660379

