# Supplementary material for: Synchrotron X-ray fluorescence microscopy unveils selenium distribution and a phloem-sink hypothesis in Neptunia amplexicaulis
Source: Plant Physiol. 2025 Aug 18;199(2):kiaf367. doi: 10.1093/plphys/kiaf367 (PMC12516699; doi:10.1093/plphys/kiaf367)
Supplement: kiaf367_Supplementary_Data [file kiaf367_supplementary_data.pdf]

SUPPLEMENTARY DATA

Synchrotron X-ray Fluorescence Microscopy unveils selenium distribution and a phloem-sink hypothesis in *Neptunia amplexicaulis*

Maggie-Anne Harvey[1,2], Peter D. Erskine[2], Hugh H. Harris[3], Katherine Pinto-Irish[2], Daryl L. Howard[4], Melody Fabillo[5], Antony van der Ent[1,2*]

[1]Laboratory of Genetics, Wageningen University and Research, The Netherlands.

[2]Centre for Mined Land Rehabilitation, Sustainable Minerals Institute, The University of Queensland, Queensland, Australia.

[3]Department of Chemistry, The University of Adelaide, Australia.

[4]Australian Synchrotron (ANSTO), Clayton, Victoria, Australia.

[5]Queensland Herbarium and Biodiversity Science, Department of Environment and Science Toowong, Queensland, Australia.

*Corresponding author: A. van der Ent (antony.vanderent@wur.nl)

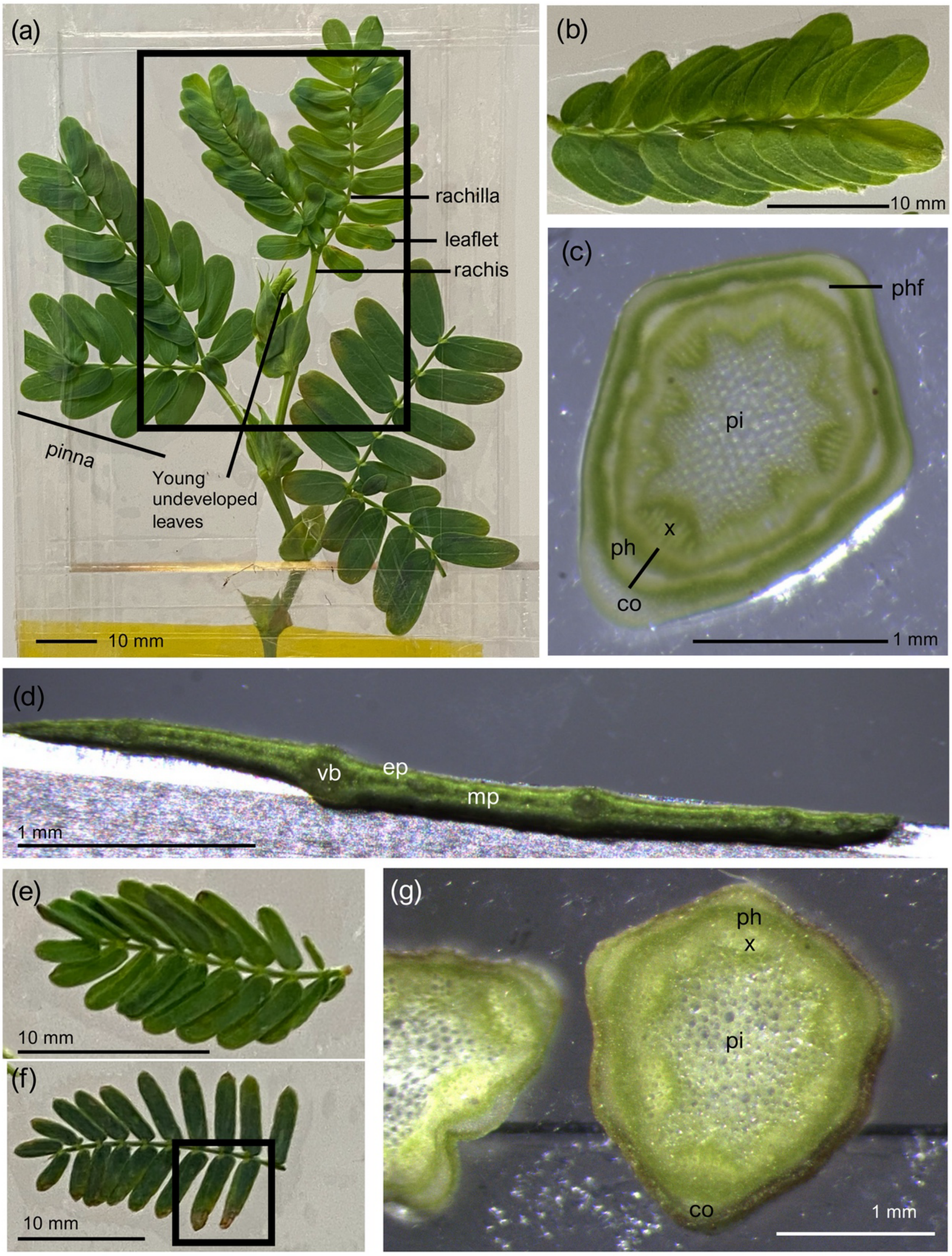

Supplementary Figure S1. Photographs and light microscope images of plant tissues used in synchrotron XFM elemental maps. *Neptunia amplexicaulis* (a) shoot tissues in figure 4a (black box shows scan area), (b) pinna in figure 4b, (c) cross sections from the same stem analysed in figure 6a and (d) type of leaf cross section analysed in figure 4c. *Neptunia heliophila* selenate dosed pinna used in (e) figure 5e and (f) figure 5c (black box shows scan area). *Neptunia heliophila* (g) cross sections from the same stem analysed in figure 6b. **co** cortex, **ep** epidermis, **mp** mesophyll, **ph** phloem, **phf** phloem fibres, **pi** pith, **vb** vascular bundle, **x** xylem.

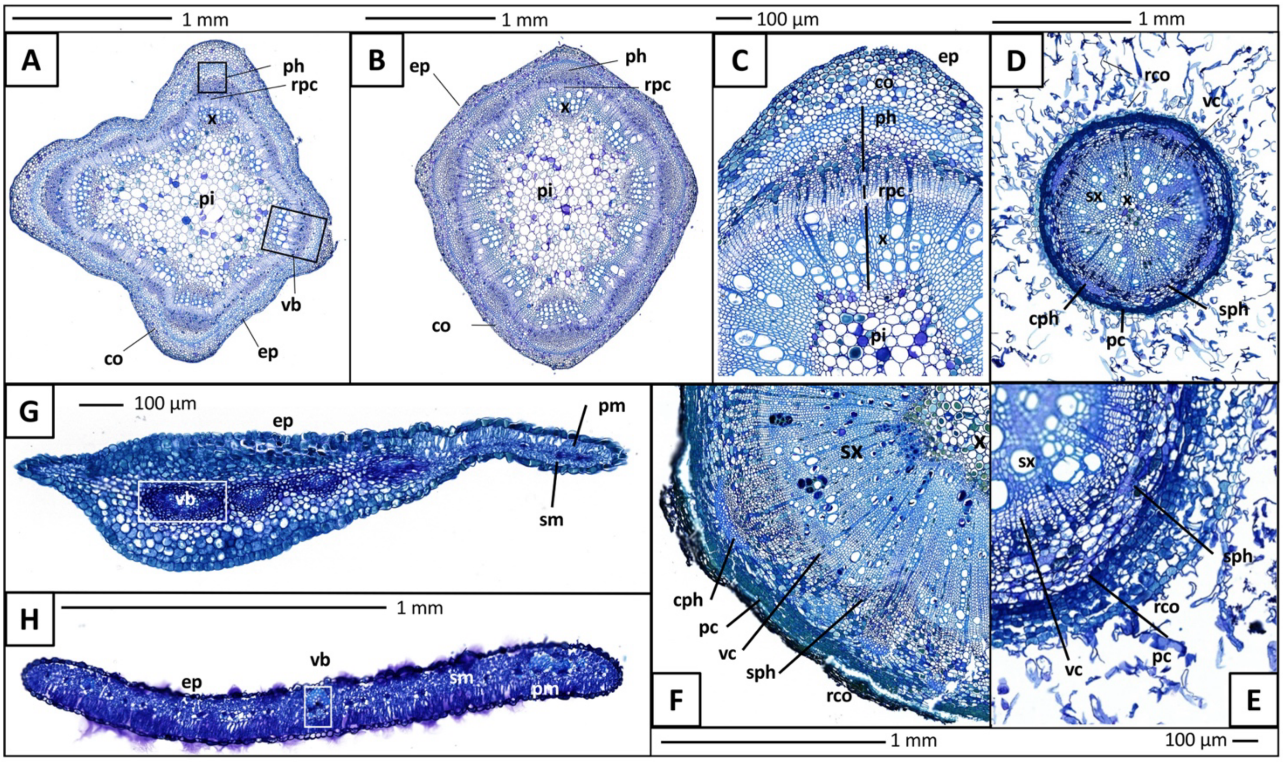

Supplementary Figure S2. Light micrographs showing Toluidine Blue stained tissue cross-sections of *Neptunia amplexicaulis* and *heliophila. Panel A: Neptunia amplexicaulis* mature stem cross section; *Panel B: Neptunia heliophila* mature stem cross section; *Panel C:* close-up of *N. amplexicaulis* basal stem cross section; *Panel D: N. amplexicaulis* hydroponic taproot cross section; *Panel E:* close up of *N. heliophila* hydroponic taproot cross section; *Panel F:* close up of *N. amplexicaulis* soil-grown taproot cross section; *Panel G: N. amplexicaulis* leaf cross section including tertiary pulvinus, *Panel H: N. heliophila* leaf cross section. Abbreviations: **ep** epidermis, **co** cortex, **vb** vascular bundle, **ph** phloem, **rpc** residual procambium, **x** xylem, **pi** pith, **rco** residual cortex, **pc** pericycle, **sph** secondary phloem, **cph** crushed primary phloem**, sx** secondary xylem, **vc** vascular cambium, **pm** palisade mesophyll, **sm** spongy mesophyll.

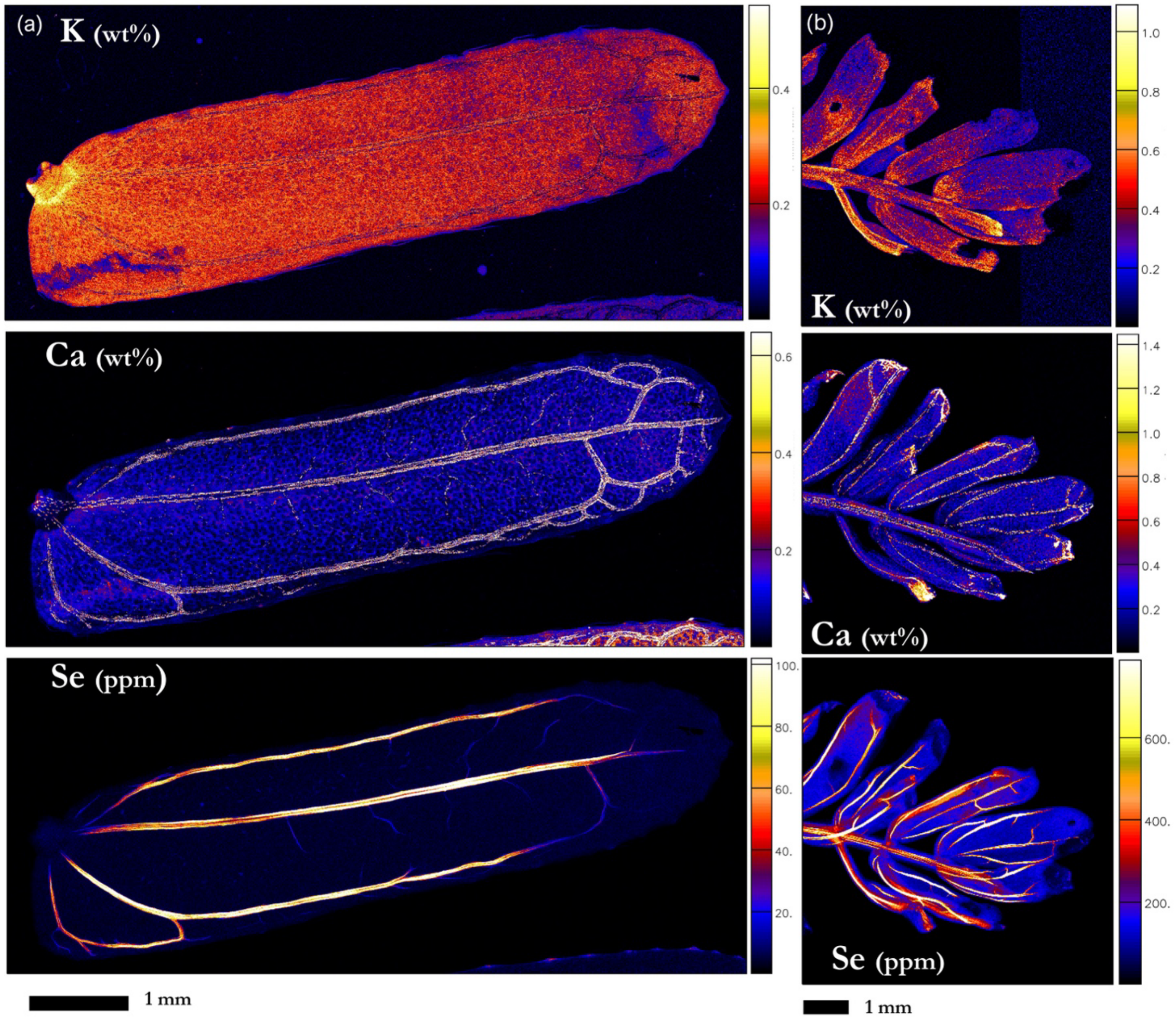

Supplementary Figure S3. Synchrotron XFM elemental maps showing the distribution of K, Ca and Se in hydrated leaf sections of *Neptunia heliophila.* (a) leaflet grown in 25 µM selenite hydroponics (r3), (b) tip of pinna with leaf damage from Se toxicity grown in 25 µM selenate hydroponics (r3). Scale bars 1 mm.

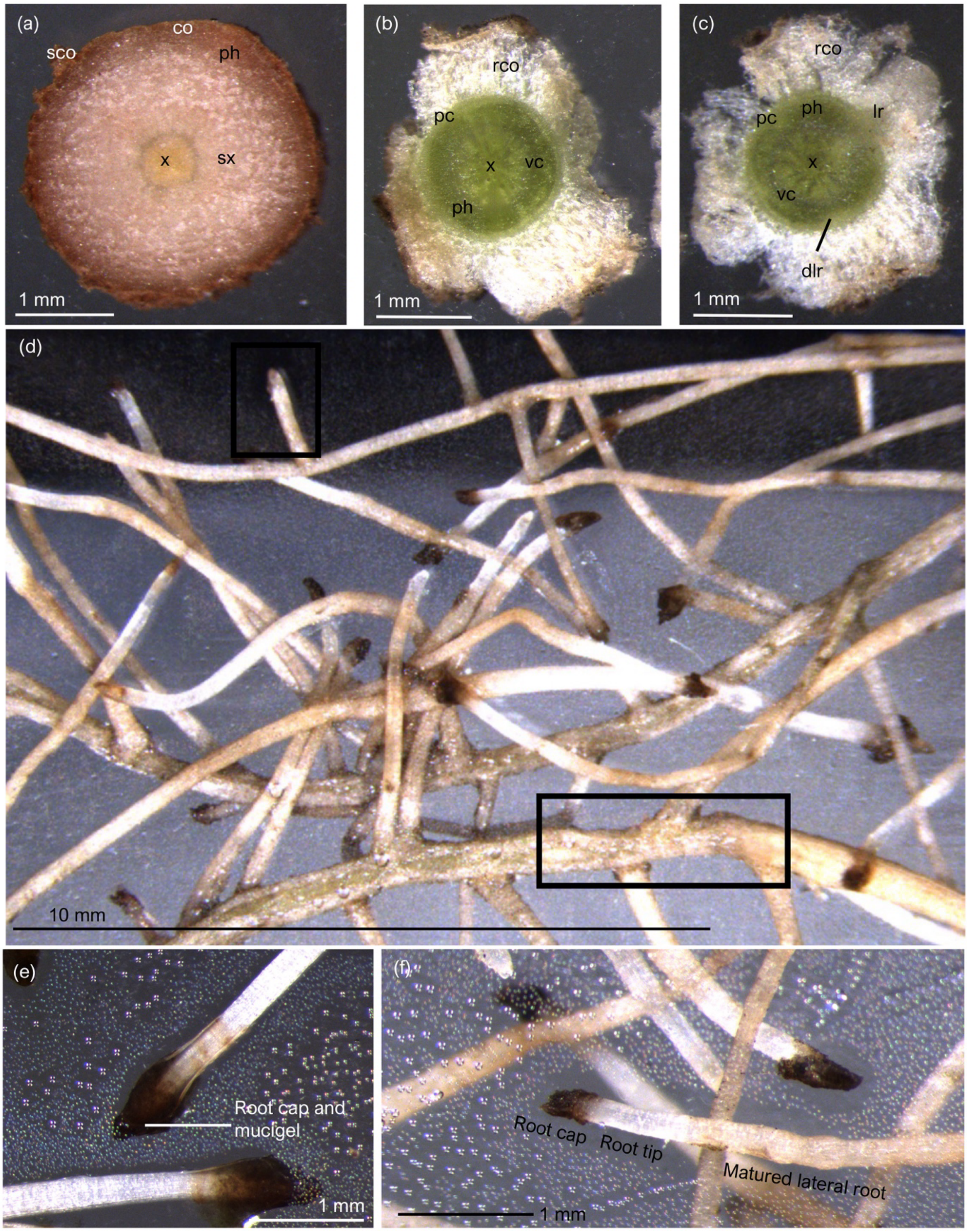

Supplementary Figure S4. Light microscope images of *Neptunia amplexicaulis* plant tissues used in synchrotron XFM elemental maps. (a) soil grown root cross section in figure 7a, (b) hydroponics root cross section in figure 7b, (c) hydroponics root cross section in figure 7c, (d) fine lateral roots in figure 8a, scan areas in figures 8b and c in black boxes (e & f) fine lateral root details. **co** cortex, **dlr** developing lateral root, **lr** lateral root, **pc** pericycle, **ph** phloem, **rco** residual cortex, **sco** sloughing cortex, **sx** secondary xylem, **vc** vascular cambium **x** xylem.

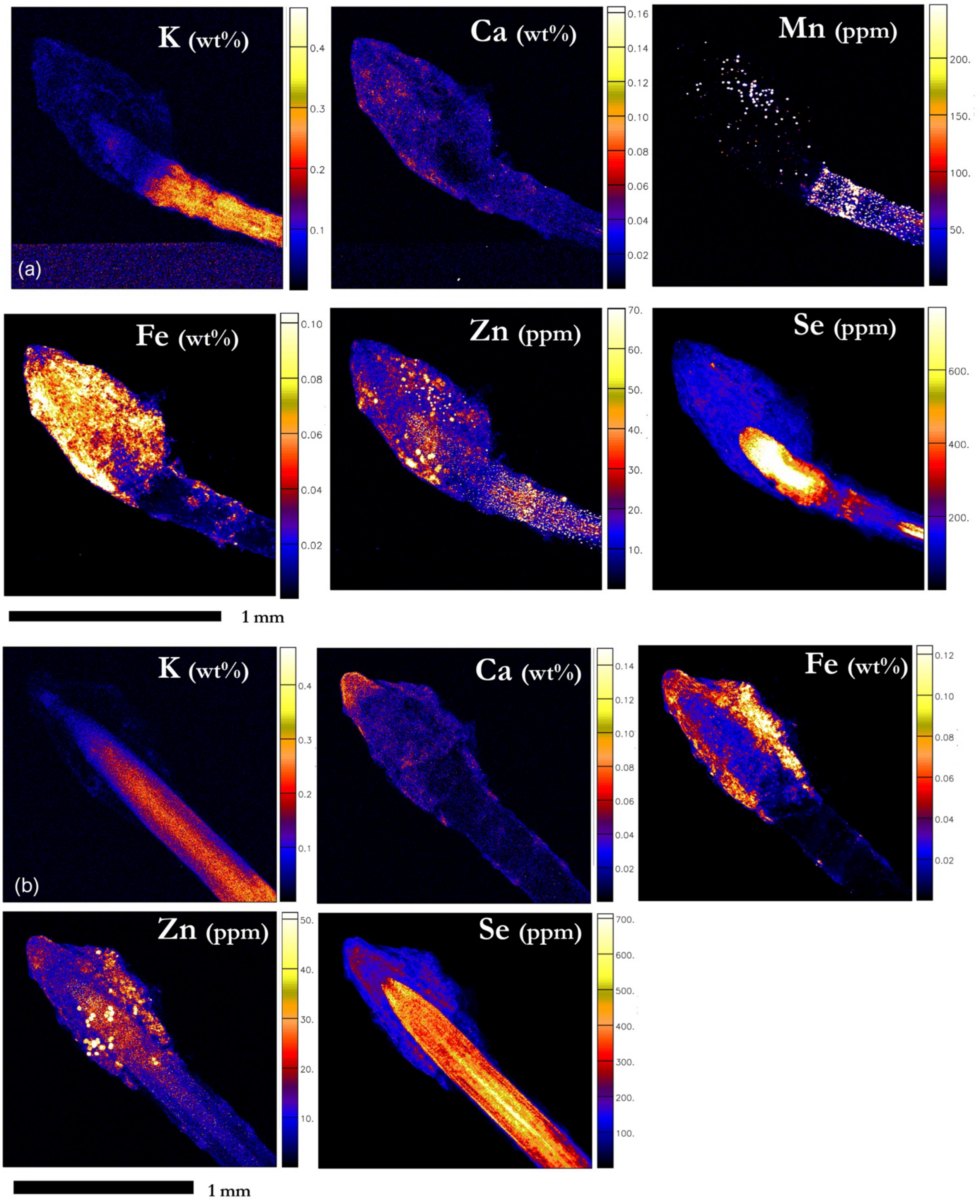

Supplementary Figure S5. Synchrotron XFM elemental maps showing the distribution of K, Ca, Fe, Mn, Zn and Se in hydrated fine roots of *N. amplexicaulis* with prominent encasing mucigel. Root tips (a) and (b) from hydroponically grown plant supplied with 75 µM selenite (r1). Scale bars 1mm.

Supplementary Table S1 Results of statistical tests run on selenium concentration by plant tissue data, subset into treatment combination, and on whole plant dry weight (dw) biomass data, subset into species. P values shown for assumption tests: Shapiro-Wilks test for normality, Levene's test for homogeneity of variances, and the same tests performed on data where the Se concentration response variable has undergone log transformation (/log). Output is the RStudio output of the chosen test.

**Treatment**	**Shapiro**	**Shapiro/log**	**Levene**	**Levene /log**	**Test**	**Output**
Neptunia amplexicaulis						
0 µM Se	>0.001	0.206	0.117	0.156	ANOVA/log	Effect DFn DFd F p p<.05 ges<br>1 TISSUE 4 55 14.404 4.16e-08 * 0.512
Selenate 25 µM	0.000213	0.0232	0.782	0.356	Kruskal-Wallis	.y. n statistic df p<br>* *<chr> <int> <dbl> <int> <dbl>*<br>1 Se196 30 15.9 4 0.00312
Selenate 50 µM	0.352	0.00165	0.0661	0.210	ANOVA	Effect DFn DFd F p p<.05 ges<br>1 TISSUE 4 25 172.122 8.37e-18 * 0.965
Selenate 100 µM	0.00850	0.669	0.161	0.0559	ANOVA/log	Effect DFn DFd F p p<.05 ges<br>1 TISSUE 4 25 25.134 1.91e-08 * 0.801
Selenite 25 µM	0.00394	0.0767	0.0679	0.0991	ANOVA/log	Effect DFn DFd F p p<.05 ges<br>1 TISSUE 4 25 30.397 2.84e-09 * 0.829
Selenite 50 µM	0.00598	0.875	0.0111	0.0660	ANOVA/log	Effect DFn DFd F p p<.05 ges<br>1 TISSUE 4 25 82.725 4.83e-14 * 0.93
Selenite 100 µM	0.292	0.143	0.00558	0.327	ANOVA/log	Effect DFn DFd F p p<.05 ges<br>1 TISSUE 4 25 25.134 1.91e-08 * 0.801
Neptunia heliophila						
0 µM Se	>0.001	>0.001	0.0113	>0.001	Kruskal-Wallis	.y. n statistic df p<br>* *<chr> <int> <dbl> <int> <dbl>*<br>1 Se196 60 27.4 4 0.0000166
Selenate 10 µM	0.359	0.306	0.615	0.276	ANOVA	Effect DFn DFd F p p<.05 ges<br>1 TISSUE 4 25 38.508 2.41e-10 * 0.86
Selenate 25 µM	0.504	0.786	0.732	0.204	ANOVA	Effect DFn DFd F p p<.05 ges<br>1 TISSUE 4 25 49.338 1.66e-11 * 0.888
Selenate 50 µM	0.843	0.256	0.189	0.534	ANOVA	Effect DFn DFd F p p<.05 ges<br>1 TISSUE 4 25 61.302 1.48e-12 * 0.907
Selenite 10 µM	0.223	0.121	0.0883	0.705	ANOVA	Effect DFn DFd F p p<.05 ges<br>1 TISSUE 4 25 41.537 1.08e-10 * 0.869
Selenite 25 µM	0.000639	0.774	0.0219	0.240	ANOVA/log	Effect DFn DFd F p p<.05 ges<br>1 TISSUE 4 25 45.026 4.5e-11 * 0.878
Selenite 50 µM	0.0946	0.000181	0.387	0.237	ANOVA	Effect DFn DFd F p p<.05 ges<br>1 TISSUE 4 25 116.432 8.87e-16 * 0.949

Biomass of whole plants (dw)						
N. amplexicaulis	0.000161	0.0559	NA	0.604	ANOVA/log	Effect DFn DFd F p p<.05 ges<br>1 O4O3_LEVEL 6 35 9.02 5.62e-06 * 0.607
N. heliophila	0.00181	0.0751	NA	0.826	ANOVA/log	Effect DFn DFd F p p<.05 ges<br>1 O4O3_LEVEL 6 35 47.852 1.96e-15 * 0.891

Supplementary Table S2. Results of post-hoc tests run on selenium concentration by plant tissue data, subset into treatment combination, and whole plant biomass by treatment combination. Output is RStudio output from each test performed, with /log indicating Se concentration data was log transformed

**Treatment**	**Test**	**Output**
Neptunia amplexicaulis		
0 µM Se	Tukey's HSD/log	term group1 group2 null.value estimate conf.low conf.high p.adj p.adj.signif<br>* *<chr> <chr> <chr> <dbl> <dbl> <dbl> <dbl> <dbl> <chr>*<br>1 TISSUE yL oL 0 -0.779 -1.52 -0.0422 0.0333 *<br>2 TISSUE yL St 0 -0.954 -1.69 -0.216 0.00512 **<br>3 TISSUE yL tr 0 0.365 -0.372 1.10 0.633 ns<br>4 TISSUE yL fr 0 0.649 -0.0879 1.39 0.109 ns<br>5 TISSUE oL St 0 -0.174 -0.911 0.563 0.963 ns<br>6 TISSUE oL tr 0 1.14 0.407 1.88 0.000502 ***<br>7 TISSUE oL fr 0 1.43 0.691 2.17 0.0000112 ****<br>8 TISSUE St tr 0 1.32 0.581 2.06 0.0000506 ****<br>9 TISSUE St fr 0 1.60 0.866 2.34 0.000000964 ****<br>10 TISSUE tr fr 0 0.284 -0.453 1.02 0.812 ns
Selenate 25 µM	Dunn's test	.y. group1 group2 n1 n2 statistic p p.adj p.adj.signif<br>* *<chr> <chr> <chr> <int> <int> <dbl> <dbl> <dbl> <chr>*<br>1 Se196 yL oL 6 6 -3.21 0.00131 0.0131 *<br>2 Se196 yL St 6 6 -2.62 0.00871 0.0871 ns<br>3 Se196 yL tr 6 6 -1.25 0.213 1 ns<br>4 Se196 yL fr 6 6 -0.295 0.768 1 ns<br>5 Se196 oL St 6 6 0.590 0.555 1 ns<br>6 Se196 oL tr 6 6 1.97 0.0491 0.491 ns<br>7 Se196 oL fr 6 6 2.92 0.00352 0.0352 *<br>8 Se196 St tr 6 6 1.38 0.168 1 ns<br>9 Se196 St fr 6 6 2.33 0.0199 0.199 ns<br>10 Se196 tr fr 6 6 0.951 0.342 1 ns
Selenate 50 µM	Tukey's HSD	term group1 group2 null.value estimate conf.low conf.high p.adj p.adj.signif<br>* *<chr> <chr> <chr> <dbl> <dbl> <dbl> <dbl> <dbl> <chr>*<br>1 TISSUE yL oL 0 -4913. -5639. -4186. 3.36e-14 ****<br>2 TISSUE yL St 0 -4378. -5104. -3651. 4.54e-14 ****<br>3 TISSUE yL tr 0 -3018. -3744. -2291. 4.91e-11 ****<br>4 TISSUE yL fr 0 -220. -946. 507. 8.99e- 1 ns<br>5 TISSUE oL St 0 535. -192. 1261. 2.27e- 1 ns<br>6 TISSUE oL tr 0 1895. 1169. 2622. 4.86e- 7 ****<br>7 TISSUE oL fr 0 4693. 3967. 5420. 3.52e-14 ****<br>8 TISSUE St tr 0 1360. 634. 2087. 9.36e- 5 ****<br>9 TISSUE St fr 0 4158. 3432. 4885. 7.25e-14 ****<br>10 TISSUE tr fr 0 2798. 2072. 3525. 2.44e-10 ****
Selenate 100 µM	Tukey's HSDl/log	term group1 group2 null.value estimate conf.low conf.high p.adj p.adj.signif<br>* *<chr> <chr> <chr> <dbl> <dbl> <dbl> <dbl> <dbl> <chr>*<br>1 TISSUE yL oL 0 -2.60 -3.57 -1.63 0.000000302 ****<br>2 TISSUE yL St 0 -1.89 -2.86 -0.921 0.0000529 ****<br>3 TISSUE yL tr 0 -0.850 -1.82 0.120 0.106 ns<br>4 TISSUE yL fr 0 0.0619 -0.908 1.03 1 ns<br>5 TISSUE oL St 0 0.708 -0.263 1.68 0.234 ns<br>6 TISSUE oL tr 0 1.75 0.779 2.72 0.000157 ***<br>7 TISSUE oL fr 0 2.66 1.69 3.63 0.000000197 ****<br>8 TISSUE St tr 0 1.04 0.0710 2.01 0.0311 *<br>9 TISSUE St fr 0 1.95 0.983 2.92 0.0000331 ****<br>10 TISSUE tr fr 0 0.912 -0.0582 1.88 0.0727 ns

Selenite 25 µM	Tukey's HSD/log	term group1 group2 null.value estimate conf.low conf.high p.adj p.adj.signi f<br>* <chr> <chr> <chr> <dbl> <dbl> <dbl> <dbl> <dbl> <chr><br>1 TISSUE yL oL 0 -2.28 -3.12 -1.44 0.000000234 ****<br>2 TISSUE yL St 0 -1.52 -2.36 -0.682 0.000146 ***<br>3 TISSUE yL tr 0 -0.302 -1.14 0.538 0.827 ns<br>4 TISSUE yL fr 0 0.375 -0.465 1.21 0.687 ns<br>5 TISSUE oL St 0 0.760 -0.0800 1.60 0.09 ns<br>6 TISSUE oL tr 0 1.98 1.14 2.82 0.00000276 ****<br>7 TISSUE oL fr 0 2.66 1.82 3.50 0.0000000133 ****<br>8 TISSUE St tr 0 1.22 0.380 2.06 0.00212 **<br>9 TISSUE St fr 0 1.90 1.06 2.74 0.00000558 ****<br>10 TISSUE tr fr 0 0.676 -0.163 1.52 0.158 ns
Selenite 50 µM	Tukey's HSD/log	term group1 group2 null.value estimate conf.low conf.high p.adj p.adj.signif<br>* <chr> <chr> <chr> <dbl> <dbl> <dbl> <dbl> <dbl> <chr><br>1 TISSUE yL oL 0 -2.53 -3.09 -1.97 7.6 e-12 ****<br>2 TISSUE yL St 0 -1.75 -2.31 -1.19 1.69e- 8 ****<br>3 TISSUE yL tr 0 -0.550 -1.11 0.0105 5.63e- 2 ns<br>4 TISSUE yL fr 0 0.400 -0.160 0.960 2.52e- 1 ns<br>5 TISSUE oL St 0 0.785 0.225 1.34 3.11e- 3 **<br>6 TISSUE oL tr 0 1.99 1.43 2.55 1.37e- 9 ****<br>7 TISSUE oL fr 0 2.93 2.37 3.50 3.16e-13 ****<br>8 TISSUE St tr 0 1.20 0.640 1.76 1.28e- 5 ****<br>9 TISSUE St fr 0 2.15 1.59 2.71 2.61e-10 ****<br>10 TISSUE tr fr 0 0.950 0.390 1.51 3.49e- 4 ***
Selenite 100 µM	Tukey's HSD/log	term group1 group2 null.value estimate conf.low conf.high p.adj p.adj.signif<br>* <chr> <chr> <chr> <dbl> <dbl> <dbl> <dbl> <dbl> <chr><br>1 TISSUE yL oL 0 -2.35 -2.66 -2.04 3.28e-14 ****<br>2 TISSUE yL St 0 -1.06 -1.37 -0.751 3 e- 9 ****<br>3 TISSUE yL tr 0 -0.148 -0.460 0.164 6.36e- 1 ns<br>4 TISSUE yL fr 0 0.922 0.610 1.23 4.83e- 8 ****<br>5 TISSUE oL St 0 1.29 0.974 1.60 5.65e-11 ****<br>6 TISSUE oL tr 0 2.20 1.89 2.51 3.30e-14 ****<br>7 TISSUE oL fr 0 3.27 2.96 3.58 3.28e-14 ****<br>8 TISSUE St tr 0 0.915 0.603 1.23 5.63e- 8 ****<br>9 TISSUE St fr 0 1.98 1.67 2.30 3.61e-14 ****<br>10 TISSUE tr fr 0 1.07 0.759 1.38 2.6 e- 9 ****
Neptunia heliophila		
0 µM Se	Dunns' test	.y. group1 group2 n1 n2 statistic p p.adj p.adj.signif<br>* <chr> <chr> <chr> <int> <int> <dbl> <dbl> <dbl> <chr><br>1 Se196 yL oL 12 12 0.187 0.852 1 ns<br>2 Se196 yL St 12 12 0.941 0.347 1 ns<br>3 Se196 yL tr 12 12 2.78 0.00550 0.0550 ns<br>4 Se196 yL fr 12 12 4.31 0.0000165 0.000165 ***<br>5 Se196 oL St 12 12 0.754 0.451 1 ns<br>6 Se196 oL tr 12 12 2.59 0.00962 0.0962 ns<br>7 Se196 oL fr 12 12 4.12 0.0000378 0.000378 ***<br>8 Se196 St tr 12 12 1.84 0.0665 0.665 ns<br>9 Se196 St fr 12 12 3.37 0.000762 0.00762 **<br>10 Se196 tr fr 12 12 1.53 0.126 1 ns
Selenate 10 µM	Tukey's HSD	term group1 group2 null.value estimate conf.low conf.high p.adj p.adj.sign if<br>* <chr> <chr> <chr> <dbl> <dbl> <dbl> <dbl> <dbl> <chr><br>1 TISSUE yL oL 0 -315. -411. -218. 0.00000000687 ****<br>2 TISSUE yL St 0 -287. -383. -191. 0.0000000411 ****<br>3 TISSUE yL tr 0 -273. -369. -177. 0.000000104 ****

		4 TISSUE yL fr 0 -68.9 -165. 27.3 0.25 ns<br>5 TISSUE oL St 0 27.7 -68.5 124. 0.914 ns<br>6 TISSUE oL tr 0 41.6 -54.6 138. 0.712 ns<br>7 TISSUE oL fr 0 246. 150. 342. 0.000000699 ****<br>8 TISSUE St tr 0 13.9 -82.3 110. 0.993 ns<br>9 TISSUE St fr 0 218. 122. 314. 0.00000524 ****<br>10 TISSUE tr fr 0 204. 108. 300. 0.0000148 ****
Selenate 25 µM	Tukey's HSD	term group1 group2 null.value estimate conf.low conf.high p.adj p.adj.signif<br>* *<chr> <chr> <chr> <dbl> <dbl> <dbl> <dbl> <dbl> <chr>*<br>1 TISSUE yL oL 0 -621. -802. -440. 2.6 e- 9 ****<br>2 TISSUE yL St 0 -741. -921. -560. 6.71e-11 ****<br>3 TISSUE yL tr 0 -688. -869. -507. 3.20e-10 ****<br>4 TISSUE yL fr 0 -659. -840. -478. 7.81e-10 ****<br>5 TISSUE oL St 0 -119. -300. 61.6 3.25e- 1 ns<br>6 TISSUE oL tr 0 -66.6 -248. 114. 8.14e- 1 ns<br>7 TISSUE oL fr 0 -37.7 -219. 143. 9.72e- 1 ns<br>8 TISSUE St tr 0 52.7 -128. 234. 9.1 e- 1 ns<br>9 TISSUE St fr 0 81.6 -99.3 263. 6.79e- 1 ns<br>10 TISSUE tr fr 0 28.9 -152. 210. 9.89e- 1 ns
Selenate 50 µM	Tukey's HSD	term group1 group2 null.value estimate conf.low conf.high p.adj p.adj.signif<br>* *<chr> <chr> <chr> <dbl> <dbl> <dbl> <dbl> <dbl> <chr>*<br>1 TISSUE yL oL 0 -1849. -2239. -1459. 2.74e-12 ****<br>2 TISSUE yL St 0 -1276. -1666. -886. 6.93e- 9 ****<br>3 TISSUE yL tr 0 -1714. -2104. -1324. 1.45e-11 ****<br>4 TISSUE yL fr 0 -1411. -1801. -1021. 8.99e-10 ****<br>5 TISSUE oL St 0 573. 183. 963. 1.88e- 3 **<br>6 TISSUE oL tr 0 134. -256. 525. 8.47e- 1 ns<br>7 TISSUE oL fr 0 437. 47.4 828. 2.25e- 2 *<br>8 TISSUE St tr 0 -439. -829. -48.5 2.21e- 2 *<br>9 TISSUE St fr 0 -136. -526. 254. 8.43e- 1 ns<br>10 TISSUE tr fr 0 303. -87.1 693. 1.84e- 1 ns
Selenite 10 µM	Tukey's HSD	term group1 group2 null.value estimate conf.low conf.high p.adj p.adj.signif<br>* *<chr> <chr> <chr> <dbl> <dbl> <dbl> <dbl> <dbl> <chr>*<br>1 TISSUE yL oL 0 -95.2 -146. -44.8 8.38e- 5 ****<br>2 TISSUE yL St 0 -90.5 -141. -40.0 1.69e- 4 ***<br>3 TISSUE yL tr 0 -51.0 -101. -0.531 4.68e- 2 *<br>4 TISSUE yL fr 0 93.6 43.2 144. 1.06e- 4 ***<br>5 TISSUE oL St 0 4.77 -45.7 55.2 9.99e- 1 ns<br>6 TISSUE oL tr 0 44.2 -6.22 94.7 1.06e- 1 ns<br>7 TISSUE oL fr 0 189. 138. 239. 4.45e-10 ****<br>8 TISSUE St tr 0 39.5 -11.0 89.9 1.79e- 1 ns<br>9 TISSUE St fr 0 184. 134. 235. 7.56e-10 ****<br>10 TISSUE tr fr 0 145. 94.2 195. 8.78e- 8 ****
Selenite 25 µM	Tukey's HSD/log	term group1 group2 null.value estimate conf.low conf.high p.adj p.adj.sign<br>if<br>* *<chr> <chr> <chr> <dbl> <dbl> <dbl> <dbl> <dbl> <chr>*<br>1 TISSUE yL oL 0 -1.25 -1.74 -0.763 0.000000695 ****<br>2 TISSUE yL St 0 -1.21 -1.70 -0.720 0.00000126 ****<br>3 TISSUE yL tr 0 -0.432 -0.922 0.0581 0.103 ns<br>4 TISSUE yL fr 0 0.591 0.101 1.08 0.0126 *<br>5 TISSUE oL St 0 0.0425 -0.448 0.533 0.999 ns<br>6 TISSUE oL tr 0 0.821 0.331 1.31 0.000409 ***<br>7 TISSUE oL fr 0 1.84 1.35 2.33 0.0000000004 ****<br>8 TISSUE St tr 0 0.778 0.288 1.27 0.00078 ***<br>9 TISSUE St fr 0 1.80 1.31 2.29 0.0000000065 ****<br>10 TISSUE tr fr 0 1.02 0.533 1.51 0.0000193 ****

Selenite 50 µM	Tukey's HSD	term group1 group2 null.value estimate conf.low conf.high p.adj p.adj.signif<br>* *<chr> <chr> <chr> <dbl> <dbl> <dbl> <dbl> <dbl> <chr>*<br>1 TISSUE yL oL 0 -368. -518. -217. 1.57e- 6 ****<br>2 TISSUE yL St 0 -387. -537. -236. 6.51e- 7 ****<br>3 TISSUE yL tr 0 -239. -390. -88.2 7.95e- 4 ***<br>4 TISSUE yL fr 0 557. 406. 707. 5.88e-10 ****<br>5 TISSUE oL St 0 -19.2 -170. 132. 9.96e- 1 ns<br>6 TISSUE oL tr 0 129. -22.2 279. 1.21e- 1 ns<br>7 TISSUE oL fr 0 924. 773. 1075. 4.12e-14 ****<br>8 TISSUE St tr 0 148. -2.97 298. 5.66e- 2 ns<br>9 TISSUE St fr 0 943. 793. 1094. 3.81e-14 ****<br>10 TISSUE tr fr 0 796. 645. 946. 2.74e-13 ****
Biomass of whole plants (DW)		
Neptunia amplexicaulis	Tukey's HSD	term group1 group2 null.value estimate conf.low conf.high p.adj p.adj.sig<br>nif<br>* *<chr> <chr> <chr> <dbl> <dbl> <dbl> <dbl> <dbl> <chr<br>>*<br>1 O4O3_LEVEL 0:0 0:25 0 1.06 0.281 1.84 0.00259 **<br>2 O4O3_LEVEL 0:0 25:0 0 1.03 0.249 1.80 0.0037 **<br>3 O4O3_LEVEL 0:0 0:50 0 1.18 0.404 1.96 0.000623 ***<br>4 O4O3_LEVEL 0:0 50:0 0 1.19 0.412 1.97 0.000567 ***<br>5 O4O3_LEVEL 0:0 0:100 0 1.74 0.965 2.52 0.000000753 ****<br>6 O4O3_LEVEL 0:0 100:0 0 0.776 -0.00137 1.55 0.0507 ns<br>7 O4O3_LEVEL 0:25 25:0 0 -0.0316 -0.809 0.746 1 ns<br>8 O4O3_LEVEL 0:25 0:50 0 0.123 -0.654 0.901 0.999 ns<br>9 O4O3_LEVEL 0:25 50:0 0 0.131 -0.646 0.908 0.998 ns<br>10 O4O3_LEVEL 0:25 0:100 0 0.684 -0.0934 1.46 0.116 ns<br>11 O4O3_LEVEL 0:25 100:0 0 -0.282 -1.06 0.495 0.913 ns<br>12 O4O3_LEVEL 25:0 0:50 0 0.155 -0.623 0.932 0.996 ns<br>13 O4O3_LEVEL 25:0 50:0 0 0.163 -0.615 0.940 0.994 ns<br>14 O4O3_LEVEL 25:0 0:100 0 0.716 -0.0618 1.49 0.088 ns<br>15 O4O3_LEVEL 25:0 100:0 0 -0.250 -1.03 0.527 0.949 ns<br>16 O4O3_LEVEL 0:50 50:0 0 0.00797 -0.769 0.785 1 ns<br>17 O4O3_LEVEL 0:50 0:100 0 0.561 -0.217 1.34 0.294 ns<br>18 O4O3_LEVEL 0:50 100:0 0 -0.405 -1.18 0.372 0.665 ns<br>19 O4O3_LEVEL 50:0 0:100 0 0.553 -0.225 1.33 0.31 ns<br>20 O4O3_LEVEL 50:0 100:0 0 -0.413 -1.19 0.364 0.645 ns<br>21 O4O3_LEVEL 0:100 100:0 0 -0.966 -1.74 -0.189 0.00722 **
Neptunia heliophila	Tukey's HSD	term group1 group2 null.value estimate conf.low conf.high p.adj p.adj.signi<br>f<br>* *<chr> <chr> <chr> <dbl> <dbl> <dbl> <dbl> <dbl> <chr>*<br>1 O4O3_LEVEL 0:0 0:10 0 0.698 -0.0233 1.42 6.32e- 2 ns<br>2 O4O3_LEVEL 0:0 10:0 0 0.503 -0.218 1.22 3.31e- 1 ns<br>3 O4O3_LEVEL 0:0 0:25 0 0.579 -0.142 1.30 1.87e- 1 ns<br>4 O4O3_LEVEL 0:0 25:0 0 -0.680 -1.40 0.0416 7.57e- 2 ns<br>5 O4O3_LEVEL 0:0 0:50 0 -1.41 -2.13 -0.686 1.12e- 5 ****<br>6 O4O3_LEVEL 0:0 50:0 0 -2.25 -2.97 -1.53 3.38e-10 ****<br>7 O4O3_LEVEL 0:10 10:0 0 -0.195 -0.916 0.526 9.78e- 1 ns<br>8 O4O3_LEVEL 0:10 0:25 0 -0.119 -0.840 0.602 9.98e- 1 ns<br>9 O4O3_LEVEL 0:10 25:0 0 -1.38 -2.10 -0.656 1.65e- 5 ****<br>10 O4O3_LEVEL 0:10 0:50 0 -2.10 -2.83 -1.38 1.81e- 9 ****<br>11 O4O3_LEVEL 0:10 50:0 0 -2.95 -3.67 -2.23 6.76e-14 ****<br>12 O4O3_LEVEL 10:0 0:25 0 0.0760 -0.645 0.797 1 e+ 0 ns<br>13 O4O3_LEVEL 10:0 25:0 0 -1.18 -1.90 -0.461 2.06e- 4 ***<br>14 O4O3_LEVEL 10:0 0:50 0 -1.91 -2.63 -1.19 1.89e- 8 ****<br>15 O4O3_LEVEL 10:0 50:0 0 -2.75 -3.47 -2.03 1.31e-12 ****<br>16 O4O3_LEVEL 0:25 25:0 0 -1.26 -1.98 -0.537 7.71e- 5 ****<br>17 O4O3_LEVEL 0:25 0:50 0 -1.99 -2.71 -1.26 7.48e- 9 ****

		18 O4O3_LEVEL 0:25 50:0 0 -2.83 -3.55 -2.11 5.32e-13 ****<br>19 O4O3_LEVEL 25:0 0:50 0 -0.727 -1.45 -0.00611 4.7 e- 2 *<br>20 O4O3_LEVEL 25:0 50:0 0 -1.57 -2.29 -0.848 1.37e- 6 ****<br>21 O4O3_LEVEL 0:50 50:0 0 -0.842 -1.56 -0.121 1.35e- 2 *

Supplementary Table S3 The whole plant biomass minimum, maximum range and mean in for each treatment. Measurements in mg dry weight (DW).

Neptunia amplexicaulis	**0 µM Se**	**Selenite 25 µM**	**Selenate 25 µM**	**Selenite 50 µM**	**Selenate 50 µM**	**Selenate 100 µM**	**Selenite 100 µM**
	349–7937<br>1885	1602–5940<br>2579	1398–3709<br>2352	1495–6116<br>2924	1985–3280<br>2639	3266–6860<br>4685	1096–2665<br>1812
Neptunia heliophila	**0 µM Se**	**Selenite 10 µM**	**Selenate 10 µM**	**Selenite 25 µM**	**Selenate 25 µM**	**Selenite 50 µM**	**Selenate 50 µM**
	2290–7513<br>3963	2390–11971<br>8476	3836–13730<br>6806	4193–10626<br>7009	748–3313<br>2101	681–1241<br>939	292–559<br>405

Supplementary Table S4. The Se concentration minimum, maximum range and mean in each plant tissue for each treatment. Selenium concentration in µg g^{-1}.

	Young leaves	Old leaves	Stems	Taproots	Fine roots
Neptunia amplexicaulis					
0 µM Se	3.11–26.9<br>9.68	1.62–3.56<br>2.51	1.19–4.28<br>2.27	3.7–15.1<br>7.24	5.21–14.9<br>8.79
Selenate 25 µM	832–3675<br>1508	67.7–943<br>317	204–943<br>485	570–2449<br>1222	839–2922<br>1495
Selenate 50 µM	4888–6631<br>5672	415–1504<br>759	902–1768<br>1294	2213–3117<br>2654	5199–5751<br>5452
Selenate 100 µM	2264–6437<br>3723	89.9–1074<br>375	193–969<br>597	940–2928<br>1632	2642–6421<br>3939
0 µM Se	1.25–7.23<br>3.66	0.869–2.42<br>1.60	0.58–2.25<br>1.44	2.71–19.9<br>7.12	5.03–20.8<br>8.75
Selenite 25 µM	277–1056<br>565	19.0–161<br>66.1	82.1–203<br>112	262–612<br>375	566–904<br>718
Selenite 50 µM	583–1639<br>1058	39.3–172<br>89.3	136–262<br>179	517–858<br>590	1082–1945<br>1524
Selenite 100 µM	1158–1499<br>1360	101–149<br>130	294–641<br>485	906–1446<br>1187	2933–3942<br>3427
Neptunia heliophila					
0 µM Se	0.335–0.54<br>0.411	0.368–0.825<br>0.507	0.389–0.594<br>0.46	0.362 - 2.28<br>1.23	1.23–5.35<br>2.54
Selenate 10 µM	388–576<br>523	160–243<br>209	157–330<br>236	201–313<br>250	365–518<br>454
Selenate 25 µM	802–1220<br>981	223–464<br>359	135–368<br>240	138–414<br>293	235–425<br>322
Selenate 50 µM	1930–2625<br>2229	277–554<br>380	411–1360<br>953	325–776<br>515	477–1092<br>818
0 µM Se	0.355–0.481<br>0.412	0.369–0.453<br>0.402	0.362–0.568<br>0.443	0.435–9.51<br>2.50	0.47–1.79<br>1.30
Selenite 10 µM	58.4–158<br>119	11.4–33.9<br>23.9	21.6–35.8<br>28.7	45.4–96.4<br>68.2	173–283<br>213
Selenite 25 µM	113–294<br>183	38.1–65.6<br>50.0	30.0–80.5<br>54.6	87.4–134<br>113	246–480<br>320
Selenite 50 µM	384–661	42.9–302	77.0–104	175–327	830–1164

	477	110	90.4	238	1034

Supplementary Table S5. Average elemental concentrations (S, K, Ca, Zn and Se) of tissues from the synchrotron XFM elemental maps of whole plant areas from Figures 4, 5 and 8. Analysis conducted using GEOPIXE, outlining the tissue of interest. Elemental concentrations in μg g^{-1}. Blank space indicates no output received per element per analysed area.

FIGURE	TISSUE	S	K	Ca	Zn	Se
4a	Apical meristem		2140			3810
	Youngest leaves		2000			4540
	Young basal leaflet		2210	631	39.0	341
	Older basal leaflet		1710	1010	37.8	123
4b	Basal leaflet	3410	2360	1160	46.1	513
	Apical leaflet	3510	2630	375	45.3	295
SI 3a	Leaflet whole		2230	1190	23.2	10.4
	Vein		2140	3360	23.4	104
	Lamina		2370	701	22.7	4.02
	Pulvinus		3070	1360	28.0	5.14
SI 3b	Apical leaflet whole		2710	2730	36.2	171
	Apical leaflet vein	2500	3000	4560	40.3	620
	Apical leaflet lamina	<252	2810	2250	35.6	115
	Rachilla		3750	4740	46.3	290
5a	Third basal leaflet		2550	1420	22.6	13.6
	Apical leaflet		2860	557	22.1	13.0
	Rachilla		4390	757	19.9	50.4
	Secondary pulvinus		3840	172	23.5	8.35
5b	Apical leaflet whole		3050	607	25.2	129
	Apical leaflet vein	1700	3040	1100	26.3	454
	Apical leaflet lamina		3020	430	24.0	82.0
	Rachilla		4270	557	25.1	317
5c	Right leaflet living	377	3600	4570	59.5	79.1
	Left leaflet living	293	3480	4480	60.2	81.5
	Right necrotic tissue	3880		7400	74.5	125
	Left necrotic tissue	397		8790	77.7	129
	Rachilla		5230	4640	80.8	44.3
8a	Root tip 1	9700	3030	647	105	1180
	Lateral root 1	4300	3130	514	176	397
	Root tip 2 (8c)	10600	3100	750	289	1000
	Lateral root 2	2400	3180	408	134	202
	Root tip 3 (not entire)	7400	2890	485	33.3	1240
	Lateral root 3	3700	3500	429	31.5	533
8b	Developing root 1		457		23.0	3570
	Developing root 2		2430		33.9	3780
	Developing root 3		1650		58.5	3200
	Root vascular bundle 1		1490	256	42.6	557
	Root vascular bundle 2		1940	273	28.8	539
	Lateral root vascular		2370	278	62.5	479
	Root cortex		2210	291	45.4	225
8c	High Se cap/meristem	12600	3640	863	118	1230
	Low Se cap/mucigel	3800	2250	791	99.1	252
	Elongation ground tissue	8690	2840	624	104	877
	Elongation zone vascular	12100	3040	745	109	1360
	Differentiation boundary	12800	3420	994	650	1170

Supplementary Table S6 Average elemental concentrations (K, Ca, Zn and Se) of tissues from the synchrotron XFM elemental maps of cross sections from Figures 4, 6 and 7. Analysis conducted using GEOPIXE, outlining the tissue of interest. Elemental concentrations in µg g^{-1}. Blank space indicates no output received per element per analysed area.

FIGURE	TISSUE	K	Ca	Zn	Se
4c	Vascular bundle	11600	4540	38.4	1150
	Epidermis	10800	2530	25.0	856
	Mesophyll	10100	2530	38.6	336
6a	Pith	859	68		25.1
	Xylem	611	169	6.31	14.4
	Phloem fibres	431	2350	6.18	13.8
	Phloem (closer to xylem)	673	343	9.24	16.7
	Cortex	1250	779	3.75	27.3
6b	Pith	2530	2880	5.25	4.77
	Xylem	4390	1170	21.2	9.64
	Phloem	4530	2690	25.7	13.6
	Cortex	6240	2220	12.0	10.5
7a	Primary xylem		10300	83.8	168
	Secondary xylem	616	525	18.8	583
	Phloem	602		1.09	274
	Pericycle	849			716
	Cortex	2200	7.14	9.09	152
	Sloughing cortex	1210	2290	24.9	82.5
7b	Xylem	1770	166	23.2	106
	Vascular cambium	2780	267	50.8	209
	Phloem	2090	436	27.6	140
	Pericycle	3400	222	18.9	723
	Cortex	2970	245	7.25	35.0
7c	Xylem (with rays)	2000	252	33.9	119
	Vascular cambium	3210	379	56.7	210
	Phloem (no lateral root)	2630	570	34.1	181
	Pericycle (no lateral root)	4440	292	19.8	696
	Lateral root (at pericycle)	3790	276	43.0	730
	Lateral root (whole)	3840	259	30	299
	Developing lateral root	3360	590	50.0	1490
	Cortex	3210	175	5.30	19.4
